# Electrically Tunable Epsilon-Near-Zero (ENZ) Metafilm Absorbers

**DOI:** 10.1038/srep15754

**Published:** 2015-11-09

**Authors:** Junghyun Park, Ju-Hyung Kang, Xiaoge Liu, Mark L. Brongersma

**Affiliations:** 1 Geballe Laboratory for Advanced Materials, Stanford University, Stanford, California 94305, United States

## Abstract

Enhancing and spectrally controlling light absorption is of great practical and fundamental importance. In optoelectronic devices consisting of layered semiconductors and metals, absorption has traditionally been manipulated with the help of Fabry-Pérot resonances. Even further control over the spectral light absorption properties of thin films has been achieved by patterning them into dense arrays of subwavelength resonant structures to form metafilms. As the next logical step, we demonstrate electrical control over light absorption in metafilms constructed from dense arrays of actively tunable plasmonic cavities. This control is achieved by embedding indium tin oxide (ITO) into these cavities. ITO affords significant tuning of its optical properties by means of electrically-induced carrier depletion and accumulation. We demonstrate that particularly large changes in the reflectance from such metafilms (up to 15% P) can be achieved by operating the ITO in the epsilon-near-zero (ENZ) frequency regime where its electrical permittivity changes sign from negative to positive values.

Light absorption is a fundamental optical phenomenon in which the energy of an electromagnetic wave impinging on an object is converted to other useful forms of energy, such as electronic, thermal, chemical, and mechanical. There are abundant applications that capitalise on these different types of conversion processes, including solar energy harvesting^1^,^2^,^3^, photo-detection^4^, optical imaging^5^, local heating^6^, medical treatments^7^, photo-catalysis^8^, and bio-sensing^9^,^10^. These have in common that they all benefit from enhancing the light absorption per unit volume. Stronger absorption increases performance of functional materials and enables the creation of more compact devices boasting a reduced materials cost and power consumption as well as increased speed of operation. Recent reports of near-unity light absorption in deep-subwavelength semiconductor films deposited on a metallic back reflector^11^,^12^,^13^ unveiled a tantalizing opportunity to realise ultrathin semiconductor devices. Unfortunately, there is not much flexibility in maximizing and controlling the spectral absorption properties with a given semiconductor and metal as their intrinsic optical materials properties are fixed. Such flexibility is afforded by metamaterials whose optical properties can be engineered with atomic-scale precision^2^,^14^,^15^. Nanometallic structures are an obvious building block for such metamaterials as they afford extreme light concentration and absorption^6^ per unit volume by virtue of surface plasmon excitations. Indeed, strong absorption of light waves has recently been achieved using dense arrays of plasmonic nanostructures^2^,^15^,^16^,^17^,^18^,^19^. In this work, we make the next step and create high-performance electrically-tunable metafilm absorbers. These are essentially single layer metamaterials constructed from subwavelength plasmonic building blocks whose strong absorption properties can be controlled electrically.

The topic of active metamaterials and metafilm has recently attracted significant attention^20^,^21^,^22^,^23^,^24^,^25^,^26^, and active absorption tuning in the mid-infrared has been accomplished with graphene-based metasurface devices^21^,^22^,^23^,^24^. Up to now the changes in the magnitude of the absorption per applied volt have been relatively small and relatively complex patterning of the graphene itself^21^ or metal structures placed on top of the graphene has been required^22^,^23^,^24^. The transparent conductor indium tin oxide (ITO) has also been used recently to electrically tune the dielectric environment of metallic antennas with the aim to manipulate their resonant absorption^25^. We show the significant benefits of using metal-insulator-metal (MIM) plasmonic cavities as the building blocks for the metafilm and embedding ITO as the active switching medium inside these cavities. We further demonstrate the significant benefits of operating the ITO near its epsilon-near-zero (ENZ) point, where it can be electrically switched from negative-permittivity to positive-permittivity^26^. This possibility is absent in graphene and may result in distinct designs.

## Basic Device Design and Operation

**Figure 1:**
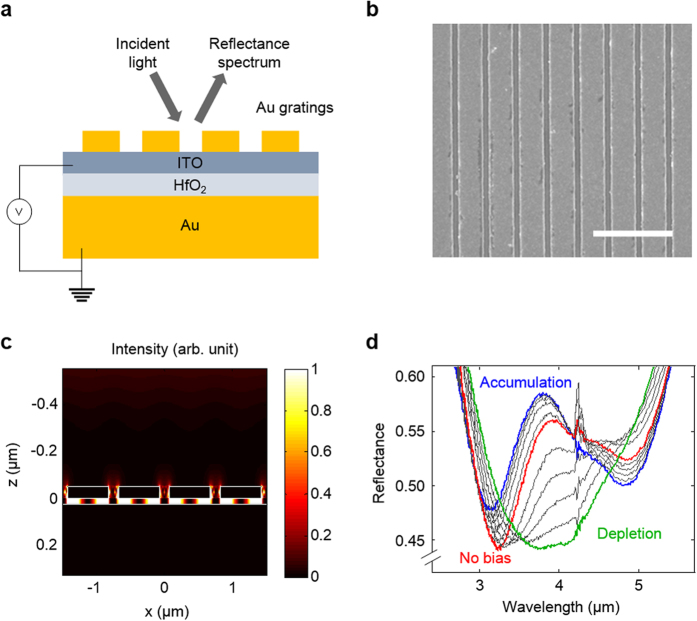
Design and performance of an electrically-tunable metafilm absorber. (**a**) Device schematic showing an electrically-tunable ITO film clamped between a HfO_2_-coated Au substrate and an array of Au strips. With positive and negative electric biases, the carrier concentration of ITO can be decreased (depletion) or increased (accumulation), leading to changes in the optical properties of the ITO. (**b**) Scanning electron microscopy (SEM) of the Au strip-array featuring strip widths of 600 nm and periods of 750 nm. Scale bar: 2 μm. (**c**) Intensity distribution in the device driven at a wavelength of 3.8 μm, which corresponds to the first-order resonance of the plasmonic cavity formed by the Au strips and the underlying substrate. (**d**) Reflectance spectra taken from the device shown in panels (**a–c**) measured using an FT-IR microscope. The red, blue, and green curves denote the reflectance spectra under 0 V for no bias, −5 V for accumulation, and +5 V for depletion, respectively. The grey curves represent reflectance spectra for intermediate voltages with an incremental step size of 1 V. The reflection spectrum signal around 4.27 μm exhibits outliers due to carbon dioxide absorption.

The proposed active metafilm absorber (Fig. 1) employs ITO to achieve electrical control over light absorption. ITO has recently emerged as a high performance plasmonic material^27^,^28^ whose optical properties can be altered substantially by tuning carrier density^29^. This has stimulated its use in new types of active devices, including waveguide modulators^30^,^31^ and optically tunable nanoantennas^32^. Figure 1a,b show a schematic and an SEM image of the proposed device in which an active ITO layer and an insulating hafnium oxide (HfO_2_) spacer are sandwiched between a subwavelength nanostrip-array and an underlying metal substrate. The strip width defines the size of the meta-atoms that make up the metafilm. The device layers were generated using standard thin film deposition processes and strip-arrays were generated by electron-beam lithography (see Methods). The thickness of the top Au, ITO, HfO_2_, and the bottom Au are 50 nm, 6 nm, 20 nm, and 50 nm, respectively. This design affords efficient electrical depletion and accumulation of carriers in the ITO layer at low voltages (~5 V). Full-field simulations indicate that normally-incident light can very effectively couple to the MIM gap plasmons^33^,^34^,^35^ between the metal strips and substrate at specific resonant wavelengths (Fig. 1c). On resonance, the coupled optical energy is effectively absorbed and dissipated in the metal and ITO regions. The electrical and optical design of the metafilm design naturally affords strong, electrically-tunable light-matter interaction and large changes in the light absorption. We will describe the design considerations for the metafilms in terms of both the geometry of the meta-atoms and the optical properties of the ITO. Both can be controlled precisely during the metafilm synthesis. We will show that particularly effective electrical modulation of light absorption can be achieved by aligning the geometric resonance of the meta-atoms with the ENZ frequency of the ITO^36^. At this frequency ITO’s permittivity changes from positive to negative values. Figure 1d shows an imprint of the electrically-controlled absorption changes for this unique case on the experimental reflectance spectra taken with a Fourier Transform infrared (FT-IR) microscope. Changes in the reflectance *R* of up to 15%P (or a modulation ratio Δ*R*/*R* = 35%) can be observed under different biasing conditions that put the ITO in a state of accumulation or depletion.

## Active Tuning of the Absorption Properties of the Individual Meta-Atoms

We start by illustrating how the resonant properties of individual plasmonic cavities can be tuned by electrical gating. These serve as the ‘meta-atoms’ that govern the properties of the metafilm. The metal strip defines the size of the cavity in which gap plasmons travel back and forth in the volume between the strip and metal substrate^37^. For individual gap plasmon resonators, it is well established that a Fabry-Pérot type resonance occurs when the round trip phase for the gap plasmons equals an integer multiple of 2π (Fig. S1.1 in Supplementary Information S1). Mathematically this is expressed as


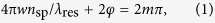


where *w* is the strip width, *n*_sp_ is the mode index of the gap plasmons, *φ* is their reflection phase, λ_res_ is the resonance wavelength, and *m* is the order of resonance. The first and second terms on the left are the propagation and the reflection phases, respectively. Equation (1) shows that electrical control over the modal properties, in particular the mode index *n*_*sp*_, would afford control over the resonance properties. Optical simulations also indicate that the reflection phase *φ* remains around 10° regardless of the bias (Supplementary Information S1), making the link between the mode index and resonance condition very simple and direct. It is well-established that the magnitude of *n*_sp_ is sensitively dependent on both the gap size and the dielectric properties of the material in the gap^33^,^34^,^35^,^38^.

**Figure 2:**
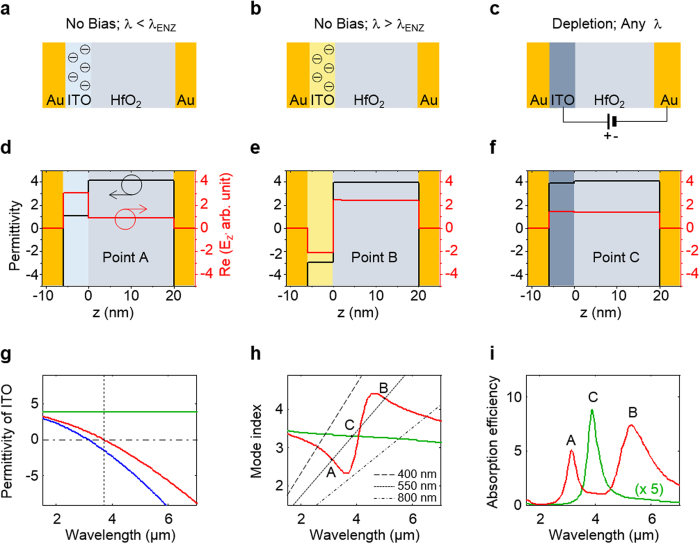
Active tuning of the meta-atom properties near the ENZ point. (**a–c**) Illustrations of the device that depict the charge distribution and optical properties of the device stack for the case (**a**) that no bias is applied and the illumination wavelength is below or (**b**) above the ENZ wavelength. (**c**) the state of the device in which a sufficiently large positive bias is applied to the ITO layer to cause full depletion. Depending on the wavelength and carrier distribution, the ITO has a positive permittivity (depicted in blue) or a negative permittivity (depicted in yellow). (**d–f**) Profiles of the permittivity (black) and the real part of the perpendicular electric field *E*_*z*_ (red) across the Au/ITO/HfO_2_/Au device stack for the biasing and illumination conditions depicted in (**a–c**). (**g**) Permittivity of the unbiased (red), depleted (green), and accumulated (blue) ITO as a function of the wavelength. The vertical black dashed line indicates the location of the ENZ wavelength of the ITO film. (**h**) Gap plasmon mode index for no bias (red) and full depletion (green). The Fabry-Pérot resonance conditions are also depicted for plasmonic cavities with strip widths of 400 nm (dashed), 550 nm (dotted), and 800 nm (dash-dotted). (**i**) Simulated absorption efficiency spectra for the 550-nm-wide strip for the case of no bias (red) and a bias that fully depletes the ITO layer (green).

To see how these quantities can be manipulated electrically, we analyse the bias-dependent optical properties of a stereotypical device that features an ITO layer with a mobile electron density of *n* = 1.47 × 10^20^/cm^3^ and a plasma frequency of *ω*_p_ = 1.02 × 10^15^  rad/s. The optical properties of ITO are modelled as a Drude metal (see Methods). Figure 2a,b depict the state of the device for the case that no bias voltage is applied and at illumination wavelengths below and above the ENZ wavelength, respectively. The red curve in Fig. 2g shows the real part of the dielectric constant for the ITO layer without bias. At shorter wavelengths, the ITO is transparent (ε > 0 and depicted as light blue in all diagrams in Fig. 2a–f) and at longer wavelengths the ITO is opaque (ε < 0 and indicated as light yellow in Fig. 2a–f). In our experiments, we dial in different, desired carrier densities for our ITO films through a choice of the deposition conditions as discussed in the Methods section.

The dependence of *n*_sp_ on wavelength for the no-bias case is shown in Fig. 2h with a standard modal analysis (see Methods). A large swing in *n*_sp_ is observed as the wavelength is tuned across the ENZ wavelength where the permittivity of ITO crosses zero. The magnitude of *n*_sp_ is significantly smaller in the spectral range where the electrical permittivity of ITO is positive than in the range where it is negative. This is intuitively explained by the strong dependence of the mode index of gap plasmons on the gap size^37^. In the negative-permittivity state of the ITO, the gap size equals the HfO_2_ thickness and in the positive-permittivity state the gap size is larger by the thickness of the ITO layer. The gap plasmon mode profiles for wavelengths of 3.1 μm (point A) and 5.0 μm (point B) shown in Fig. 2d,e illustrate these changes. The magnitude of *n*_sp_ also sensitively depends on the actual magnitude of the permittivity of ITO. Part of the high sensitivity comes from the fact that the dominant electric field for gap plasmons is normal to the metal surfaces and thus also normal to the ITO film. The required continuity of the normal component of the electric displacement field, *D*_*n*_ = *εE*_*n*_ at a boundary gives rise to substantial changes in the mode profile as the real part of epsilon crosses zero. This leads to the sizeable swing in the mode index with increasing wavelength near λ = 3.7 μm.

To learn where resonances will occur for 550-nm-wide strips, we plot the first-order (*m* = 1) Fabry-Pérot resonance condition (Eq. 1) as a dotted black line in Fig. 2h. Two absorption peaks occur in the absorption efficiency (defined by the total absorption of light per incoming photon flux on the strip-width) spectra where the red curve crosses the resonance condition at points A and B, as shown in Fig. 2i. An alternative vantage point is that the two absorption peaks result from the coupling of a materials (plasma) resonance of the ITO and a geometric resonance of the plasmonic cavity. When the strip width is sufficiently decreased/increased, these resonances are detuned and only a single resonant absorption peak is observed for one specific resonance order. This can be seen from an analysis of the first-order (*m* = 1) Fabry-Pérot resonance condition for 400-nm-wide (dashed) and 800-nm-wide (dash-dotted) strips in Fig. 2h. These situations will be shown in experiment later, but first we analyse the impact of applying a bias for the 550-nm-wide strips.

The application of a positive/negative bias to the ITO layer causes depletion or accumulation of mobile carriers. The DC electrostatics and free carrier distribution in the ITO layer is modelled using a stair-case approximation (See Methods). The application of a sufficiently large positive bias to the ITO layer depletes this layer from mobile electrons (Fig. 2c). This effectively moves the plasma frequency to zero. As a result, the ITO exhibits a positive wavelength-independent permittivity as shown by the solid green curves (Fig. 2g). The spectral dependence of the mode index in this bias state is much simpler and shown by the green curve in Fig. 2h. In this state, the Fabry-Pérot resonance condition is only met at one spectral location (Point C). This fact results in one peak in the absorption spectrum (green curve) shown in Fig. 2i. In Supplementary Information S2 we continue to analyse the effect of depletion and accumulation for various grating widths. From this analysis, it is clear that the largest electrically-induced change in the absorption spectrum occurs when the plasma frequency (materials resonance) is aligned with the geometric resonance frequency of the plasmonic strip cavities. These changes can be induced in a continuous fashion as the bias voltage is increased and a depletion region is created with a width proportional to the generated DC electric field (See Supplementary Information S3).

## Construction of the Metafilm from Active Building Blocks

**Figure 3:**
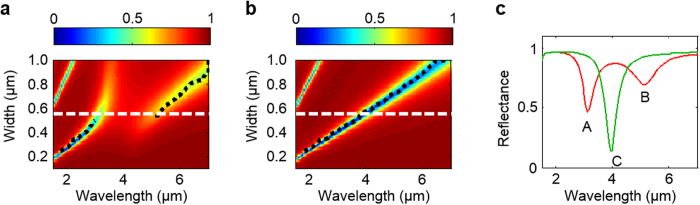
Construction of the metafilm from active building blocks. (**a,b**) Reflectance map for a metafilm constructed from strip cavities spaced at a period of 1200 nm as functions of wavelength and strip width for (**a**) unbiased and (**b**) depleted cases. The black dotted lines show the wavelength with the maximum absorption efficiency of individual strip cavities as displayed in Fig. 2i. The metafilm’s resonant properties in the subwavelength regime closely follow those of the individual building blocks. The horizontal dashed white lines denote a case of the strip width of 550 nm (Fig. 3c). (**c**) Reflectance spectrum of a strip-array featuring strip width of 550 nm and spacing of 650 nm (period: 1200 nm) for unbiased (red) and depleted (green) cases. Points A, B, and C here correspond to points A, B, and C in Fig. 2h,i, respectively.

To show how the resonant properties of the individual building blocks can be encoded into the optical properties of the metafilm, we simulate the reflectance spectra of metafilms composed of an array of strip cavities with a period of 1200 nm and for various strip widths. These results were used to create maps of the reflectances versus illumination wavelength and stripe width for the case of no-bias (Fig. 3a) and for depletion (Fig. 3b). We first note that an increase in strip width tends to lead to a longer resonance wavelength. This can be ascribed to the increased roundtrip phase for the gap-plasmons due to an increased path length in traversing the strip cavity. The other branches on the left top corners of the map are the third order Fabry-Pérot resonance. Secondly, the wavelengths featuring the maximum absorption efficiency in individual single strip cavities (as in Fig. 2i) are calculated for each strip width and plotted as black dotted lines in Fig. 3a,b. They show that the reflectance dips of the metafilm are spectrally aligned with the absorption efficiency peaks of the single strip plasmonic cavities in Fig. 2i. Further numerical investigations show that the spectral position of the resonance wavelength is hardly affected by the spacing between individual strip cavities in the subwavelength period regime (Supplementary Information S4). This result demonstrates that the resonant response of the individual meta-atom can effectively be encoded into the reflectance properties of the metafilm.

Bearing in mind that the strip width is the governing design factor for the resonance wavelength, we now seek to identify the wavelength regime in which the highest absorption modulation can be achieved. In the no-bias case (Fig. 3a), there is an anti-crossing of the plasmonic cavity resonance related to the strip width due to the material resonance around the ENZ wavelength (~4 μm). As we apply the positive bias to fully deplete the ITO (Fig. 3b), the material resonance around the ENZ wavelength disappears and a linear relationship between the strip width and the resonance wavelength is observed. From these simulations it is clear that the strongest modulation occurs when the strip width is designed to overlap the plasmonic cavity resonance with the material resonance around the ENZ wavelength. In Fig. 3c, we plot a reflectance spectrum from the 550 nm-width and 1200 nm-spacing strip cavity arrays for no-bias (red) and for depleted (green) cases, which correspond to the horizontal dashed lines in Fig. 3a,b. The Points A, B, and C correspond to those in Fig. 2h,i. In addition to the clear agreement between the reflectance dips (Fig. 3c) and the absorption peaks (Fig. 2i), we observe the large modulation around the ENZ wavelength (Point C). This observation points at a powerful design strategy for metafilm absorber layers that can highly leverage our knowledge of individual gap plasmon polariton resonators (Supplementary Information S5).

## Experimental Demonstration of Active Metafilm Absorption Tuning

**Figure 4:**
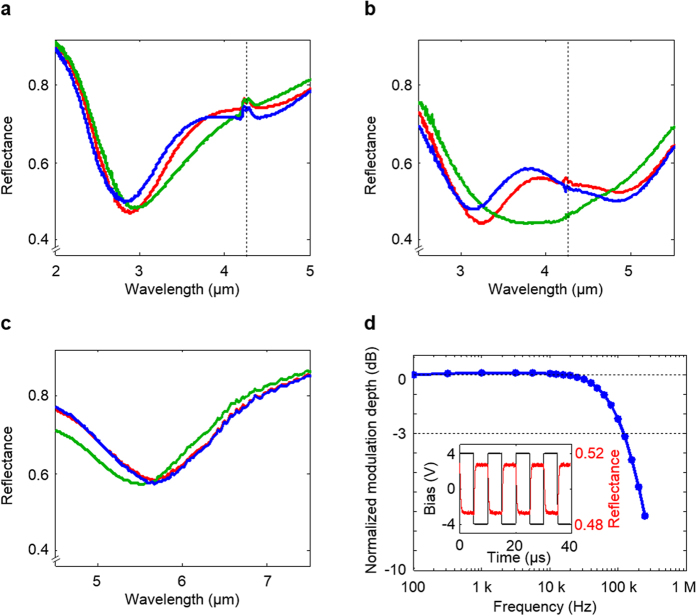
Experiment demonstration of tuning. (**a**) Reflectance spectrum for the circumstance where the grating width is 490 nm and the period is 640 nm. The resonant wavelength is shorter than the ENZ wavelength (*λ*_res_ < *λ*_ENZ_, where *λ*_res_ for accumulation, depletion, and no-bias are 2.81 μm, 3.01 μm, and 2.88 μm, respectively, and *λ*_ENZ_ is 4.28 μm). The reflection spectrum signal around 4.27 μm exhibits a feature linked to carbon dioxide absorption. (**b**) Grating width is 600 nm (*λ*_res_ ≈ *λ*_ENZ_, where *λ*_res_ for accumulation are 3.14 μm and 4.86 μm (double dips), that for depletion is 3.88 μm (single dip), and those for no-bias are 3.25 μm and 4.87 μm (double dips). *λ*_ENZ_ is 4.28 μm and the period is 750 nm. (**c**) Grating width is 720 nm (*λ*_res_ > *λ*_ENZ_, where *λ*_res_ for accumulation, depletion, and no-bias are 5.67 μm, 5.51 μm, and 5.70 μm, respectively, and *λ*_ENZ_ is 4.28 μm) and the period is 920 nm. The red, green, and blue curves denote the no bias, the positive electric bias for depletion (+5 V), and the negative electric bias for accumulation accumulation (−5 V), respectively. The vertical black dashed line indicates the location of the ENZ wavelength of the ITO film. The reflectance in the depletion operation (red to green curves) around the ENZ wavelength (*λ*_res_ ≈ *λ*_ENZ_) exhibits the largest reflectance changes. (**d**) Frequency characteristics. The normalized modulation depth features a 3 dB cut-off frequency around 125 kHz. The inset in Fig. 4d shows the temporal reflectance changes (red) upon voltage changes (black) for a sample with the grating width of 600 nm and a period 750 nm. The positive voltage (+4 V) forms the depletion region, giving rise to the decreased reflectance, whereas the negative voltage (−4 V) causes carrier accumulation and slightly raises the reflectance.

Devices with metal gratings featuring different widths are fabricated and the reflectance spectra in states of depletion and accumulation are collected (Supplementary Information S6). Figure 4a–c show selected spectral reflectance measurement obtained from samples with Au subwavelength gratings featuring widths of 490 nm, 600 nm, and 720 nm, respectively. The ITO layer in these samples was measured to have an ENZ wavelength *λ*_ENZ_ = 4.3 μm (Supplementary Information S7), as denoted by the vertical dashed lines. These strips produce plasmonic cavity resonances at shorter, similar, and longer wavelengths than *λ*_ENZ_. The spectra obtained for no-bias (red), depletion (green, +5 V), and accumulation (blue, −5 V) are shown in each figure. The depletion and accumulation width for these voltages and our specific device geometries are found to be similar and close to 1.5 nm (See Methods). Larger depletion layer widths could have been obtained if the breakdown field of the oxide layer could be improved beyond the current value of 3.5 MV/cm (Supplementary Information S8). Simulated spectra based on a rigorous coupled wave analysis^39^ (RCWA) for these geometries and biasing conditions are shown in Supplementary Information S6, in which the simulation results show good overall agreement with the experimental ones.

Figure 4a shows a reflectance dip at 2.9 μm for the narrow strip-array (*w* = 490 nm; *λ*_res_ < *λ*_ENZ_) when no bias is applied to the device (red curve). The application of a positive/negative bias can be used to form a depletion/accumulation region that produces redshift/blueshift of the reflectivity dip (green/blue curves). These shifts are explained by a bias-induced increase/decrease in the mode index. Figure 4c shows that for the wide strip array (*w* = 720 nm; *λ*_res_ > *λ*_ENZ_) a resonance dip occurs at 5.7 μm (red curve). The depletion gives rise to a blueshift of the resonance, which is opposite to the depletion-induced redshift for the metafilm with the narrow strips (green curve). This can be explained considering Fig. 2h, which shows that the change in the mode index upon depletion of the ITO is in opposite directions for wavelength above and below the ENZ wavelength. No significant changes in the peak position and depth are seen upon accumulation of the wide strip array (blue curve, Supplementary Information S6). This is because the emergence of an accumulation layer that contributes to an increase of the magnitude of the negative permittivity of the ITO layer. As a result the field distribution of the gap plasmon is not significantly changed, and the mode index exhibits only a slight decrease.

As predicted in Fig. 3c, spectral alignment of the plasmonic cavity resonance with the materials resonance at the *λ*_ENZ_ causes two dips in the reflectance spectra (red curve in Fig. 4b with *w* = 600 nm; *λ*_res_ ≈ *λ*_ENZ_). As the depletion region is formed under the positive bias, those two reflectance dips merge into a single dip (green curve). This results in a substantial decrease in the experimentally measured reflectance at *λ* = 3.8 μm, close to *λ*_ENZ_. By combining the depletion and accumulation operations over this wavelength range, one can maximise the modulation efficiency up to 15% P. The measurements and simulations show the benefits of aligning the optical materials and geometric cavity resonances in achieving the largest modulation efficiency. This modulation efficiency is not as high as those shown in simulations (Fig. 3c), which assumed full depletion could be achieved with a high performance gate oxide such as HfO_2_.

To demonstrate the dynamic control of the depletion/accumulation action on the metafilm absorption, we analysed the temporal changes in the reflectance signal upon application of time-varying bias to the device with the strip width of 600 nm and the array period of 750 nm with the wavelength of 3.00 μm to 3.50 μm (see Methods.). The inset in Fig. 4d shows the applied voltage (black) for a square pulse with a max/min voltage of +4 V/−4 V and the resultant reflectance (red). The frequency response in Fig. 4d indicates a 3 dB cut-off frequency around 125 kHz, which is in reasonable agreement with theoretical estimation of 172 kHz based on the resistivity of the ITO layer and device capacitance (see Methods.). The modulation speed would be further improved with a lower capacitance that is achievable by decreasing the area and a smaller resistance that can be realized by patterning both ITO and Au strips and by gating through Au strips.

## Conclusion

We have demonstrated electrically-controlled metafilm absorbers that leverage the unique tunable optical properties of ITO and the excellent field concentrating properties of plasmonic cavities. Particularly strong absorption modulation could be achieved when the ITO is operated in the ENZ spectral range, where a sign change of the permittivity from negative value to positive value can be induced electrically. The current work can easily be extended to plasmonic structures with more complex geometries and Supplementary Information S9 discusses the polarisation-independent absorber structures based on metal disc arrays. This work finds direct application in a range of the optoelectronic devices that require active control over light absorption and/or emission. These include essentially flat components for signal modulation, wavefront manipulation dynamic control over thermal emission, sensing, camouflage, and solar energy harvesting to adapt to changes in the environment (e.g. changes in the position of the sun in the sky). For many such applications, the 125 kHz modulation rate of absorption would be of significant practical value. The work also adds to the recent flurry of activity on active plasmonics and metamaterials.

## Methods

### Fabrication and optical characterisation

The metafilms are realised by first depositing a 50-nm-thick Au layer with a 5-nm-thick Ti adhesion layer atop of a smooth quartz substrate by e-beam evaporation. A 20-nm-thick insulating HfO_2_ layer is then deposited by atomic layer deposition (ALD) and followed by deposition of a 6-nm-thick ITO film using DC magnetron sputtering^40^. Finally, 50-nm-thick top Au gratings and discs are generated by conventional liftoff techniques. Patterning of each layer is performed using standard optical and electron beam lithography. Reflectance measurements are made using a FT-IR microscope (Thermo Scientific). A polariser is used to analyse the 1-D gratings under transverse magnetic (TM) polarised light, where the electric field is perpendicular to the gratings. An objective lens with a numerical aperture of 0.58 with the maximum incident angle 35° was used.

To evaluate the frequency response of the metafilms, we placed a band pass filter with transmission band from 3.00 μm to 3.50 μm in the beam path of the FT-IR set-up. We used an InSb infrared photodetector (IS-2.0, InfraRed Associates, Inc.) with a preamplifier (INSB-1000, InfraRed Associates, Inc.) that has the electrical bandwidth 1 MHz. The modulated signal under sinusoidal wave of +4 V/−4 V was processed by using a lock-in amplifier (7265, AMTEK Signal Recovery, Inc.). The capacitance of the ITO/HfO_2_/Au configuration was measured around 185 pF, and the resistance of the ITO around 5.0 kΩ, which leads to a 3 dB cut-off frequency ~172 kHz.

### Modelling and simulation

ITO is modelled as a Drude metal with a permittivity given by *ε* = *ε*_inf_ − *ω*_p_^2^/(*ω*^2^ + *i*Γ*ω*). *ε*_inf_ is the infinite frequency permittivity, *ω*_p_ is the plasma frequency, *ω* is the angular frequency of light, and Γ is the collision frequency. The plasma frequency *ω*_p_ of the ITO film is extracted by measuring the reflectance from the ITO/HfO_2_/Au stack and normalizing the result with that from HfO_2_/Au (Supplementary Information S7), and is obtained as 1.02  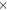 10^15^ rad/s. The collision frequency Γ of the ITO is obtained by investigating the difference of the reflectance spectra of metallic gratings on the ITO/HfO_2_/Au and HfO_2_/Au, and is obtained as 2.6 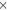
10^14^ rad/s. These parameters are used throughout this Article unless specified separately. In the theoretical analysis of the mode index change and the resultant absorption efficiency and reflectance spectra in Figs 2 and 3, the collision frequency Γ of the ITO is set to 1.0  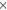 10^14^ rad/s in order to effectively visualise the change of the mode index. For *ε*_inf_ = 3.9, *ω*_p_ = 1.02 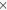
10^15^ rad/s, and Γ = 1.0 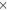 10^14^ rad/s, the ENZ wavelength is obtained as *λ*_ENZ_ = 3.7 μm. The magnitude of *ω*_p_ is determined by the carrier density as *ω*_p_^2^ = *ne*^2^/(*m*_e_^*^ε_0_)^40^, where *n* is the electron density, *e* the electron charge, and *ε*_0_ the free-space permittivity. *m*_e_^*^ denotes the effective mass of the electrons. In the ITO, our previous work reported *m*_e_^*^ ≈ 0.45 *m*_*e*_, where *m*_*e*_ is the electron mass in free space^41^.

The refractive index of HfO_2_ for the infrared regime is obtained by ellipsometry (J. A. Woollam, M-2000 and IR-VASE, Supplementary Information S10). The optical properties of Au are captured by a Drude model with *ε*_inf_ = 9, *ω*_p_ = 1.38  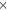 10^16^ rad/s, and Γ = 1.09  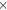 10^14^ rad/s^42^. The refractive index of the quartz substrate is set to 1.43.

The current across the 20 nm-thick-insulating layer remained negligibly small values and we found currents in the nA range for voltages in the 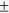
7 V range (Supplementary Information S8), beyond which the dielectric breakdown occurs and a steep increase in the current is observed. The DC permittivity of the insulating layer (HfO_2_) is measured as 16, and the depletion width at 5 V across 20 nm reaches around 1.5 nm (Supplementary Information S8).

In the modal analysis, the top and bottom Au are set to be semi-infinite. This assumption is plausible owing to the very large magnitude of the negative real part of the electric permittivity of Au in the infrared regime. The characteristic equation for the configuration is obtained by using the general approach for an arbitrary step-index multilayer waveguide, which is based on the field continuity condition at each boundary between neighbouring layers^35^,^40^.

For full-field electromagnetic simulation, the RCWA was used for simulation^39^. The number of harmonics was 151, which were proven to be enough to generate converged data.

## Additional Information

**How to cite this article**: Park, J. *et al.* Electrically Tunable Epsilon-Near-Zero (ENZ) Metafilm Absorbers. *Sci. Rep.* **5**, 15754; doi: 10.1038/srep15754 (2015).

## Supplementary Material

Supplementary Information

## References

[b1] Atwater H. A. & Polman A. Plasmonics for improved photovoltaic devices. Nature Mater. 9, 205–213 (2010). 20168344 10.1038/nmat2629

[b2] Hägglund C. *et al.* Self-assembly based plasmonic arrays tuned by atomic layer deposition for extreme visible light absorption. Nano Lett. 13, 3352–3357 (2013). 23805835 10.1021/nl401641v

[b3] Kim S. J. *et al.* Light trapping for solar fuel generation with Mie resonances. Nano Lett. 14, 1446−1452 (2014). 24524658 10.1021/nl404575e

[b4] Cao L. *et al.* Engineering light absorption in semiconductor nanowire devices. Nature Mater. 8, 643–647 (2009). 19578337 10.1038/nmat2477

[b5] Zhang R. *et al.* Chemical mapping of a single molecule by plasmon-enhanced Raman scattering. Nature 498, 82–86 (2013). 23739426 10.1038/nature12151

[b6] Baffou G. & Quidant R. Thermo-plasmonics: Using metallic nanostructures as nano-sources of heat. Laser Photon. Rev. 7, 171–187 (2013).

[b7] Hirsch L. R. *et al.* Nanoshell-mediated near-infrared thermal therapy of tumors under magnetic resonance guidance. Proc. Natl. Acad. Sci. USA 100, 13549–54 (2003). 14597719 10.1073/pnas.2232479100 PMC263851

[b8] Christopher P., Xin H. & Linic S. Visible-light-enhanced catalytic oxidation reactions on plasmonic silver nanostructures. Nature Chem. 3, 467–472 (2011). 21602862 10.1038/nchem.1032

[b9] Anker J. N. *et al.* Biosensing with plasmonic nanosensors. Nature Mater. 7, 442–453 (2008). 18497851 10.1038/nmat2162

[b10] Adato R. & Altug H. In-situ ultra-sensitive infrared absorption spectroscopy of biomolecule interactions in real time with plasmonic nanoantennas. Nature Comm. 4, 2154 (2013). 10.1038/ncomms3154 PMC3759039 23877168

[b11] Kats M. A., Blanchard R., Genevet P. & Capasso F. Nanometre optical coatings based on strong interference effects in highly absorbing media. Nature. Mater. 12, 20−24 (2013). 23064496 10.1038/nmat3443

[b12] Dotan H. *et al.* Resonant light trapping in ultrathin films for water splitting. Nature Mater. 12, 158−164 (2013). 23142836 10.1038/nmat3477

[b13] Park J. *et al.* Omnidirectional near-unity absorption in an ultrathin planar semiconductor layer on a metal substrate. Am. Chem. Soc. Photon. 1, 812–821 (2014).

[b14] Landy N., Sajuyigbe S., Mock J. J., Smith D. R. & Padilla W. J. Perfect metamaterial absorber. Phys. Rev. Lett. 100, 207402 (2008). 18518577 10.1103/PhysRevLett.100.207402

[b15] Aydin K., Ferry V. E., Briggs R. M. & Atwater H. A. Broadband polarization-independent resonant light absorption using ultrathin plasmonic super absorbers. Nature Comm. 2, 517 (2011). 10.1038/ncomms1528 22044996

[b16] Søndergaard T. *et al.* Plasmonic black gold by adiabatic nanofocusing and absorption of light in ultra-sharp convex grooves. Nature Comm. 3, 969 (2012). 10.1038/ncomms1976 22828629

[b17] Liu N., Mesch M., Weiss T., Hentschel M. & Giessen H. Infrared perfect absorber and its application as plasmonic sensor. Nano Lett. 10, 2342–2348 (2010). 20560590 10.1021/nl9041033

[b18] Hao J. *et al.* High performance optical absorber based on a plasmonic metamaterial. Appl. Phys. Lett. 96, 251104 (2010).

[b19] Wu C., Neuner B. III & Shvets G. Large-area wide-angle spectrally selective plasmonic absorber. Phys. Rev. B 84, 075102 (2011).

[b20] Zheludev N. I. & Kivshar Y. S. From metamaterials to metadevices. Nature Mater. 11, 917–924 (2012). 23089997 10.1038/nmat3431

[b21] Jang M. S. *et al.* Tunable Large resonant absorption in a midinfrared graphene Salisbury screen. Phys. Rev. B 90, 165409 (2014).

[b22] Yao Y. *et al.* Electrically tunable metasurface perfect absorbers for ultrathin mid-infrared optical modulators. Nano Lett. 14, 6526–6532 (2014). 25310847 10.1021/nl503104n

[b23] Davidian N. *et al.* Electrical switching of infrared light using graphene integration with plasmonic Fano resonant metasurfaces. Am. Chem. Soc. Photon. 2, 216–227 (2015).

[b24] Emani N. K. *et al.* Electrically tunable damping of plasmonic resonances with graphene. Nano Lett. 12, 5202–5206 (2012). 22950873 10.1021/nl302322t

[b25] Yi F. *et al.* Voltage tuning of plasmonic absorbers by indium tin oxide. Appl. Phys. Lett. 102, 221102 (2013).

[b26] Jun Y. C. *et al.* Epsilon-near-zero strong coupling in metamaterial-semiconductor hybrid structures. Nano Lett. 13, 5391–5396 (2013). 24124754 10.1021/nl402939t

[b27] Boltasseva A. & Atwater H. A. Low-loss plasmonic metamaterials. Science 331, 290–291 (2011). 21252335 10.1126/science.1198258

[b28] Naik G. V., Liu J., Kildishev A. V., Shalaev V. M. & Boltasseva A. Demonstration of Al:ZnO as a plasmonic component for near-infrared metamaterials. Proc. Natl. Acad. Sci. USA 109, 8834–8838 (2012). 22611188 10.1073/pnas.1121517109 PMC3384131

[b29] Feigenbaum E., Diest K. & Atwater H. A. Unity-order index change in transparent conducting oxides at visible frequencies. Nano Lett. 10, 2111–2116 (2010). 20481480 10.1021/nl1006307

[b30] Sorger V. J., Lanzillotti-Kimura N. D., Ma R.-M. & Zhang X. Ultra-compact silicon nanophotonic modulator with broadband response. Nanophotonics 1, 17–22 (2012).

[b31] Vasudev A. P., Kang J.-H., Park J., Liu X. & Brongersma M. L. Electro-optical modulation of a silicon waveguide with an “epsilon-near-zero” material. Opt. Express 21, 26387–26397 (2013). 24216861 10.1364/OE.21.026387

[b32] Abb M., Albella P., Aizpurua J. & Muskens O. L. All-optical control of a single plasmonic nanoantenna-ITO hybrid. Nano Lett. 11, 2457–2463 (2011). 21542564 10.1021/nl200901w

[b33] Zia R., Selker M. D., Catrysse P. B. & Brongersma M. L. Geometries and materials for subwavelength surface plasmon modes. J. Opt. Soc. Am. A 21, 2442–2446 (2004). 10.1364/josaa.21.002442 15603083

[b34] Park J. & Lee B. An approximate formula of the effective refractive index of the metal–insulator–metal surface plasmon polariton waveguide in the infrared region. J. Jap. Appl. Phys. 47, 8449–8451 (2008).

[b35] Park J. *et al.* Trapping light in plasmonic waveguides. Opt. Express 18, 598–623 (2010). 20173880 10.1364/OE.18.000598

[b36] Maas R., Parsons J., Engheta N. & Polman A. Experimental realization of an epsilon-near-zero metamaterial at visible wavelengths. Nature Photon. 7, 907–912 (2013).

[b37] Søndergaard T. & Bozhevolnyi S. I. Strip and gap plasmon polariton optical resonators. Phys. Stat. Sol. (B) 245, 9–19 (2008).

[b38] Pors A., Albrektsen O., Radko I. P. & Bozhevolnyi S. I. Gap plasmon-based metasurfaces for total control of reflected light. Sci. Rep. 3, 2155 (2013). 23831621 10.1038/srep02155 PMC3703605

[b39] Kim H., Park J. & Lee B. Fourier Modal Method and its Applications in Computational Nanophotonics. (CRC Press, Boca Raton, 2012).

[b40] Saleh B. E. A. & Teich M. C. Fundamentals of Photonics. 2nd Ed. (Wiley-Interscience, Hoboken, 2007).

[b41] Liu X. *et al.* Quantification and impact of nonparabolicity of the conduction band of indium tin oxide on its plasmonic properties. Appl. Phys. Lett. 105, 181117 (2014).

[b42] Cai W. & Shalaev V. Optical Metamaterials: Fundamentals and Applications. (Springer, New York, 2010).

