# Supplementary material for: Electrically Tunable Epsilon-Near-Zero (ENZ) Metafilm Absorbers
Source: Sci Rep. 2015 Nov 9;5:15754. doi: 10.1038/srep15754 (PMC4637893; doi:10.1038/srep15754)
Supplement: Supplementary Information [file srep15754-s1.pdf]

Supplementary Information for
"Electrically Tunable Epsilon-Near-Zero (ENZ) Metafilm Absorbers"

Junghyun Park,[§] Ju-Hyung Kang,[§] Xiaoge Liu, Mark L. Brongersma[*]

Geballe Laboratory for Advanced Materials, Stanford University, Stanford, California 94305, United States

*Corresponding author: brongersma@stanford.edu

S1. Fabry-Pérot resonance condition for a plasmonic strip cavity

The strong absorption in the proposed metafilm devices finds its origin in the excitation of resonating gap plasmons in plasmonic strip cavities that make up the metafilm. The spectral location of the resonances is determined by a Fabry-Pérot condition; the resonance occurs when the overall phase pickup for gap plasmons in a cavity round trip equals an integer multiple of 2π (Fig. S1.1). The propagation phases are denoted by the symbols ① and ③ in Fig. S1.1, and are dependent on the mode index of the gap plasmon. The main text and Supplementary Information S2 show how this mode index can change with the illumination wavelength and the applied electrical bias. In this section, we discuss the magnitude of reflection phase pickup (② and ④ in Fig. S1.1).

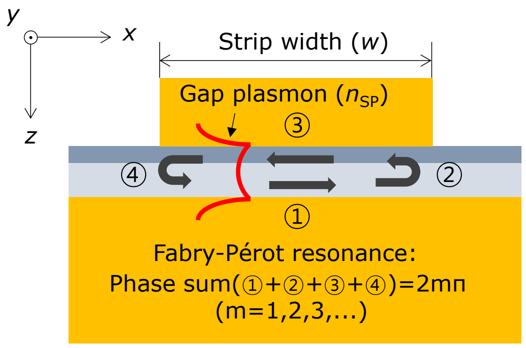

Figure S1.1. Schematic diagram showing a cross section of a plasmonic strip cavity. It shows an Au substrate and strip (yellow) that clamp ITO (dark grey) and HfO_2 (light grey) layers. It illustrates the Fabry-Pérot resonance condition. A resonance occurs when the overall phase pickup for gap plasmons in making a cavity round trip equals an integer multiple of 2π.

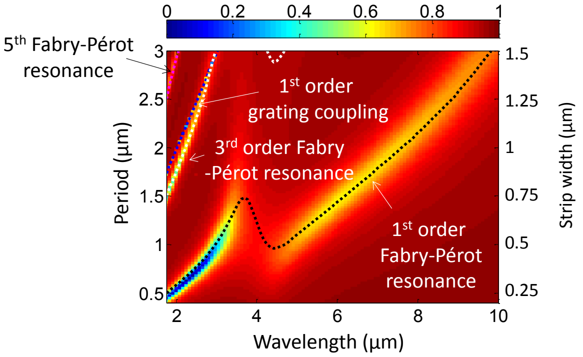

Figure S1.2. Reflectance map as a function of wavelength and period with the filling ratio 0.5. The strip widths corresponding to each period are provided along the right axis. Around the wavelength of 4 μm, the strip resonance interacts with the ITO materials resonance, giving rise to the "swing" in the reflectance map. The dashed black, white, and magenta lines correspond to first, third, and fifth order Fabry-Pérot resonances, respectively. The dotted blue line indicates the condition for grating coupling a normally-incident wave to the plane of the metafilm.

In order to understand the magnitude of the reflection phases, the component ② and ④ in Fig. S1.1, we calculated map of the reflectance versus the array period and illumination wavelength. A metal strip with a filling fraction of 0.5 (duty cycle is 1:1) was used (Fig. S1.2) and a larger period thus implies a wider strip width. In this example, the full complexity of the HfO_2/ITO stack in the plasmonic cavity is considered. The ENZ wavelength of ITO was taken to be 3.7 μm. Based on the Fabry-Pérot resonance condition provide in equation (1) of the main text ($4\pi w n_{sp}/\lambda + 2\varphi = 2m\pi$), a linear scaling of the resonant width with the illumination wavelength would be expected. However, the rapid changes on the mode index in near the ENZ wavelength produce a swing in the resonant strip width with increasing wavelength.

The Fabry-Pérot resonance condition for the 1st, 3rd, and 5th Fabry-Pérot resonance are plotted with dashed lines in the Fig.S1.2. The numerical studies based on the rigorous coupled-wave analysis (RCWA) shows that the constant reflection phase of 10° exhibits good agreement with the calculated reflection maps. In addition, the reflection phase does not vary significantly across the considered wavelength range. This is because the reactive energy stored in the near-field out of the end facet of the resonator does not significantly change as the wavelength in all cases is substantially larger than the gap width of the gap resonator.

S2. Depletion and accumulation operation in various strip widths

In Fig. 2 of the main text, we presented the impact of an applied bias on the light absorption for the case of (i) carrier depletion and (ii) $\lambda_{res} \approx \lambda_{ENZ}$, where λ_{res} is the gap plasmon resonance wavelength of a strip cavity and λ_{ENZ} the epsilon-near-zero wavelength of the ITO. In this section, we provide a comprehensive investigation for other cases. It will be shown that operation in the $\lambda_{res} \approx \lambda_{ENZ}$ regime is of critical importance to maximise the absorption modulation ratio.

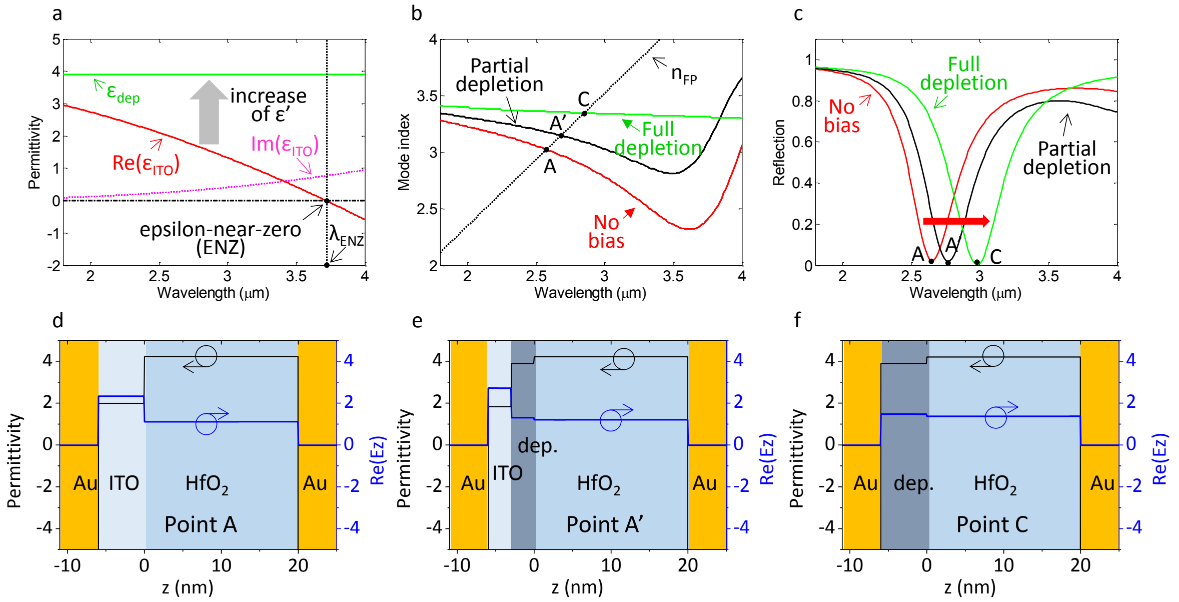

Figure S2.1. **a,** Permittivity of the ITO and the depletion region for a narrow, 400-nm-wide strip for which $\lambda_{res} < \lambda_{ENZ} = 3.7$ μm. **b,** Mode index of the gap plasmons for no bias, partial depletion, and full depletion. The dashed black line denotes the Fabry-Pérot mode index n_{FP} for width 400 nm. The crossing points of the dashed and solid lines are locations where optical resonances occur for different biasing conditions and are labeled with capital letters. **c,** Full-field simulation result for the strip width 400 nm and period 600 nm. **d,e,f,** Permittivity (black, the left *y*-axis) and the real part of the perpendicular electric field (blue, the right *y*-axis) distribution for Points A, A', and C in Figs. S2.1 **b** and **c**, respectively.

Figure S2.1 shows the relevant optical properties to understand the changes in the reflectance spectra for the depletion operation in the $\lambda_{res} < \lambda_{ENZ}$ regime. The changes in the spectra are similar in nature to the shorter wavelength reflectance dip of the double reflectance dips in the $\lambda_{res} \approx \lambda_{ENZ}$ regime, which is covered in the main text. The difference is the strip width is smaller, so that there is only a single reflectance dip, instead of double dips. Due to the narrow strip width the resonance wavelength lies below the ENZ wavelength and the

dielectric constant of the ITO for the no-bias condition is positive, as can be seen as the solid red curve in Fig. S2.1 a. As a positive bias is applied to the ITO layer, a depletion region is formed by pushing the electrons out of the ITO layer. Consequently, the gap plasmons will experience a raised dielectric constant in the gap and an increased mode index (Fig. S2.1 b). Fig. S2.1 b also shows that the Fabry-Pérot resonance condition (dashed line) line. The crossing points of the solid and dashed lines, where the Fabry-Pérot resonance condition is met, indicate the plasmonic resonance is expected to occur at increasingly long wavelengths as the ITO layer is depleted. The change in the mode profile shown in Figs. S2.1 d,e, and f are consistent with this analysis and show the increased overlap with the increasingly high permittivity ITO layer.

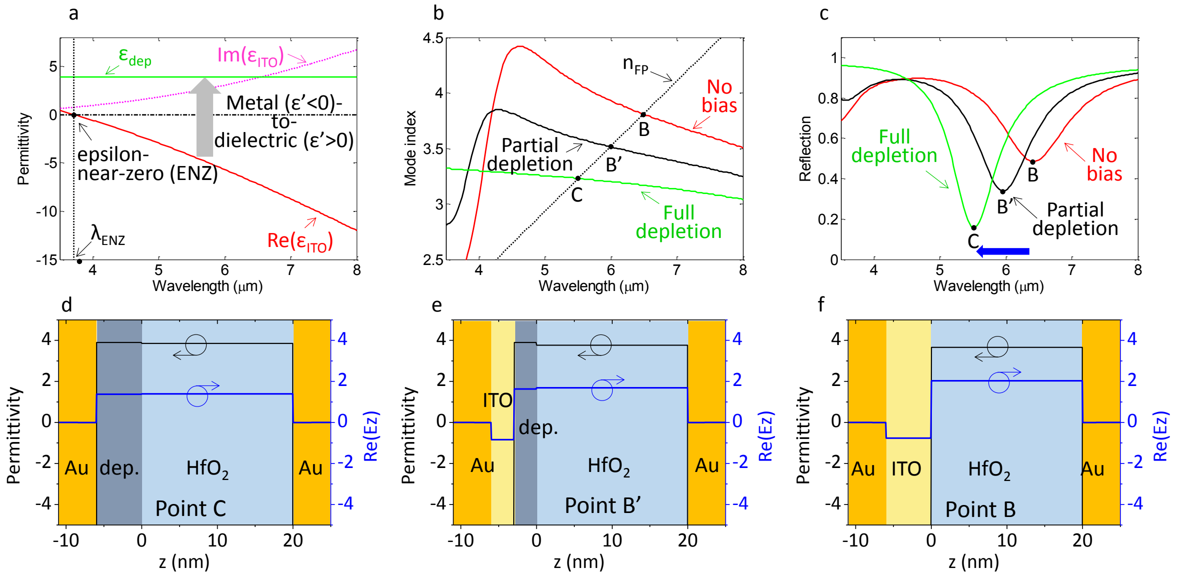

Figure S2.2. **a,** Permittivity of the ITO and the depletion region for an 800-nm-wide strip for which $\lambda_{res} > \lambda_{ENZ}$. **b,** Mode index of the gap plasmons for the cases of no bias, partial depletion, and full depletion. The dashed black line denotes the Fabry-Pérot mode index n_{FP} for the considered strip width of 800 nm. **c,** Full-field simulation of the reflectance for a metafilm with 800-nm-wide strips spaced at a period of 1000 nm. **d,e,f,** Permittivity (black, the left *y*-axis) and the real part of E_z (blue, the right *y*-axis) distribution for Points C, B', and B in Figs. S2.2 **b** and **c**, respectively.

As a next step, we discuss the depletion operation for wider strip cavities for which $\lambda_{res} > \lambda_{ENZ}$. There is also a single reflectance dip as for the case that $\lambda_{res} < \lambda_{ENZ}$, but the spectral shift in the reflectance dip is in the opposite direction. This can again be understood from the changes in ITO's permittivity shown in Fig. S2.2 a. At wavelengths longer than λ_{ENZ}, the real

part of the permittivity is negative (Fig. S2.2 f). As a result, the real part of the electric field perpendicular to the ITO layer (E_z) changes its sign upon crossing the interface between the ITO and the HfO_2 layers that features a positive permittivity. The resonance wavelength is found when the Fabry-Pérot condition is satisfied (Point B in Fig. S2.2 b) and can be also be found from the simulated reflectance spectrum (Point B in Fig. S2.2 c). The reflectance minimum is not as deep as and a bit broader than the other strip widths. This can be ascribed to the strong materials loss of ITO in this wavelength regime (see the dashed magenta curve in Fig. S2.2 a). Under the depletion operation, the electrical permittivity ITO changes from negative value to positive value. This increases the effective wavelength of the gap plasmons due to the extension of the effective core width (Fig. S2.2 e). Consequently, the resonance wavelength displays the blueshift from Point B to Point B' in Figs. S2.2 b and S2.2 c. As the applied voltage increases, the ITO layer is fully depleted, forming the blueshifted resonant wavelength (Point C in Figs. S2.2 b and S2.2 c).

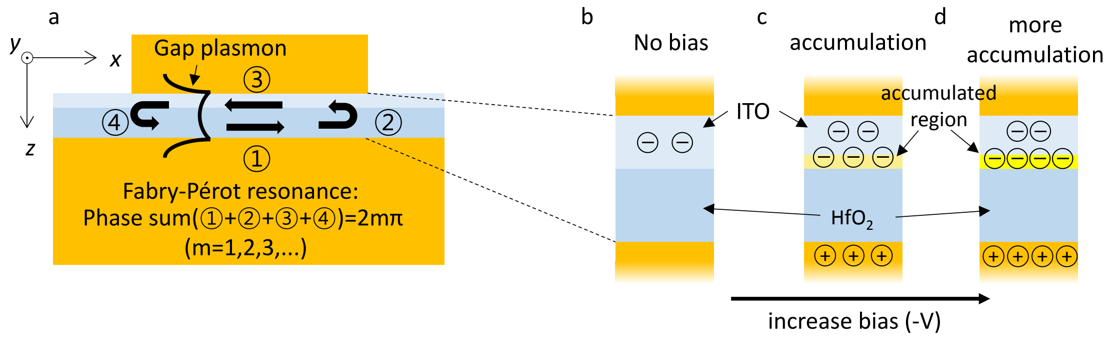

Figure S2.3. a, Schematic diagram of a unit cell for the Fabry-Pérot resonance of the gap plasmons. **b,** Zoom-in illustration of the ITO and HfO_2 layer in the no bias case. **c,** Negative electric bias to the ITO induces the increase of the electron density at the interface between the ITO and HfO_2 layer, forming the accumulation region. **d,** As the negative bias is increased, higher concentration is obtained.

Next, we consider what happens when the devices are operated in accumulation. (See Fig. S2.3). If no bias is applied, the electron concentration in the ITO layer is constant (See Fig. S2.3 b). As a negative bias is applied to the ITO layer, additional electrons are pulled into to the layer and agglomerate near the interface between the ITO and HfO_2 layers, forming an accumulation layer (Fig. S2.3 c). The electron concentration further increases linearly with increasing negative bias (Fig. S2.3 d).

Figure S2.4 shows the impact of carrier accumulation on the optical properties for a middle-

sized strip width for which $\lambda_{res} \approx \lambda_{ENZ}$. For wavelengths below λ_{ENZ}, the ITO layer behaves as a dielectric (Figs. S2.4 e for Point A). As the charge carrier concentration increases at the interface between the ITO and HfO_2 layers, a thin layer is produced in which the plasma frequency is increased. As a result the dielectric constant is decreased in this short wavelength regime. This causes a local field enhancement in the accumulation region. This field enhancement is particularly large for the wavelength that matches λ_{ENZ} of the accumulation layer (see Fig. S2.4 d Point C). This in turn leads to slight decrease of the mode index (Fig. S2.4 b), and a concomitant slight blueshift of the resonance wavelength (Fig. S2.4 c). We note that the direction of the shift is opposite to the case where the ITO is depleted. Consequently, one can utilise both the blueshift and the redshift of the resonance wavelength under the accumulation and depletion to achieve a higher modulation efficiency.

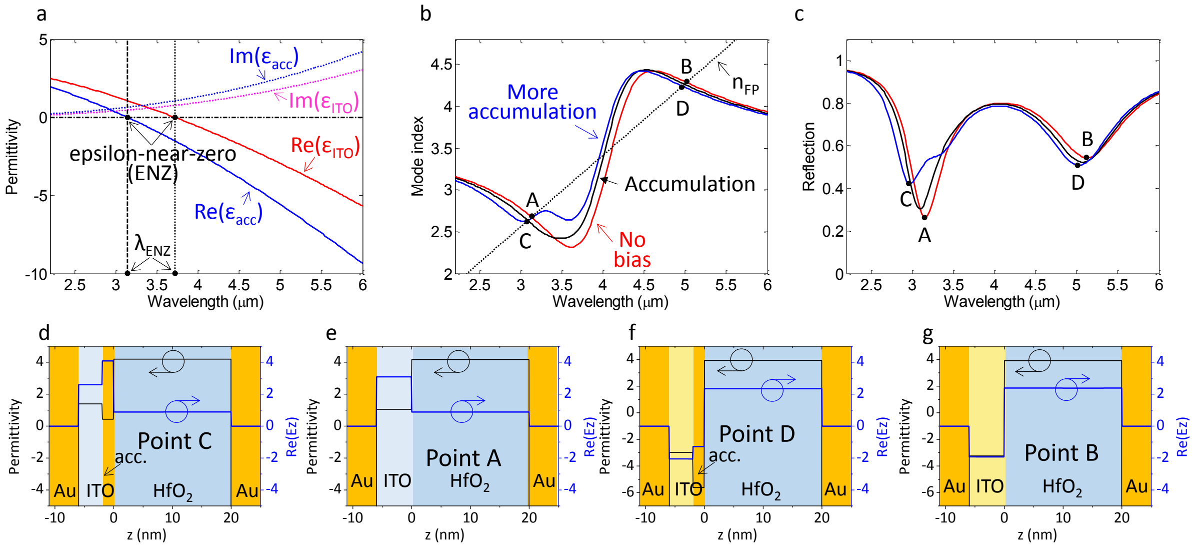

Figure S2.4. a, Permittivity of the ITO and the accumulation region for a middle-sized strip width ($\lambda_{res} \approx \lambda_{ENZ}$ regime). **b,** Mode index of the gap plasmons for the cases of no bias, accumulation, and strong accumulation. The dashed black line denotes the Fabry-Pérot mode index n_{FP} for a strip width of 550 nm. **c,** Full-field simulation results of the reflectance of a metasurface with 550-nm-wide strips spaced by 700 nm. **d,e,f,** Permittivity (black, the left y-axis) and the real part of E_z (blue, the right y-axis) distribution for Points C, A, D, and B in Figs. S2.4 **b** and **c**, respectively.

Let us next examine the functional behavior for the wavelength regime longer than the ENZ wavelength. The ITO layer in this regime features a negative sign of the real part of the permittivity, and its value decreases further when the ITO film is brought into a state of accumulation by application of a negative bias (Fig. S2.4 a). The field profile of the gap

plasmons does not change significantly (Figs. S2.4 f and g), since the effective dielectric core width that the gap plasmons experience remains almost the same. This is because the magnitude of the negative permittivity of ITO is increased. As a result, the resonance wavelength only shows a slight blueshift (Fig. S2.4 c). It can be seen that the amount of spectral shift achievable in accumulation mode operation is negligible compared to that under the depletion operation. In addition, both operations of depletion and accumulation exhibit blueshift. Therefore, the modulation efficiency that can be achieved in this regime is mostly determined by the depletion operation.

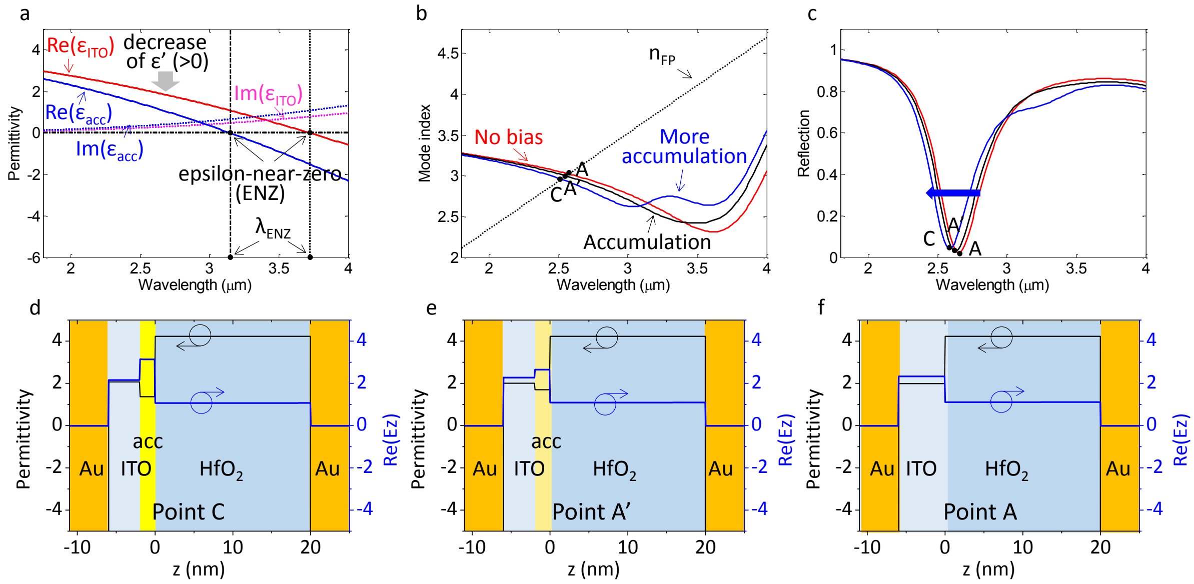

Figure S2.5. a, Permittivity of the ITO and the accumulation region for a narrow strip width for which $\lambda_{res} < \lambda_{ENZ}$. **b,** Mode index of the gap plasmons for the cases of no bias, accumulation, and more accumulation. The dashed black line denotes the Fabry-Pérot mode index n_{FP} for a strip width of 400 nm. **c,** Full-field simulation result for the reflectance of a metafilm constructed from 400-nm-wide strips spaced at a 600 nm period. **d,e,f,** Permittivity (black, the left y-axis) and the real part of E_z (blue, the right y-axis) distribution for Points C, A', and A in Figs. S2.5 **b** and **c**, respectively. The blue arrow in Fig. S2.5 c denotes the blueshift of the resonance wavelength.

Let us consider the accumulation operation for narrow strips for which $\lambda_{res} < \lambda_{ENZ}$. In Fig. S2.5 a, the increase of the plasma frequency coming from the accumulation leads to a decrease in the real part of the ITO permittivity. The change of a large and positive permittivity to a small, but still positive permittivity of the core region induces a decreased mode index (Fig. S2.5 f to e for accumulation, and e to d for more accumulation). The wavelength at which the

Fabry-Pérot resonance condition is satisfied (again indicated with capital letters) moves toward the shorter wavelength (Fig. S2.5 b). The numerical investigation demonstrates that the resonance wavelength exhibits the blueshift (Fig. S2.5 c).

As the last step, we consider the case of a wide strip width for which $\lambda_{\mathrm{res}} > \lambda_{\mathrm{ENZ}}$. The accumulation mode operation lowers the permittivity (Fig. S2.6 a). As mentioned above in Fig. S2.4, this does not induce significant changes in the mode profile of the gap plasmons (Figs. S2.6 d, e, and f). Therefore the wavelength at which the Fabry-Pérot resonance condition is met remains almost the same (Fig. S2.6 b). This can also be confirmed by the full-field simulation of the reflectivity in Fig. S2.6 c, which shows a negligible spectral shift in the location of the reflectance dip.

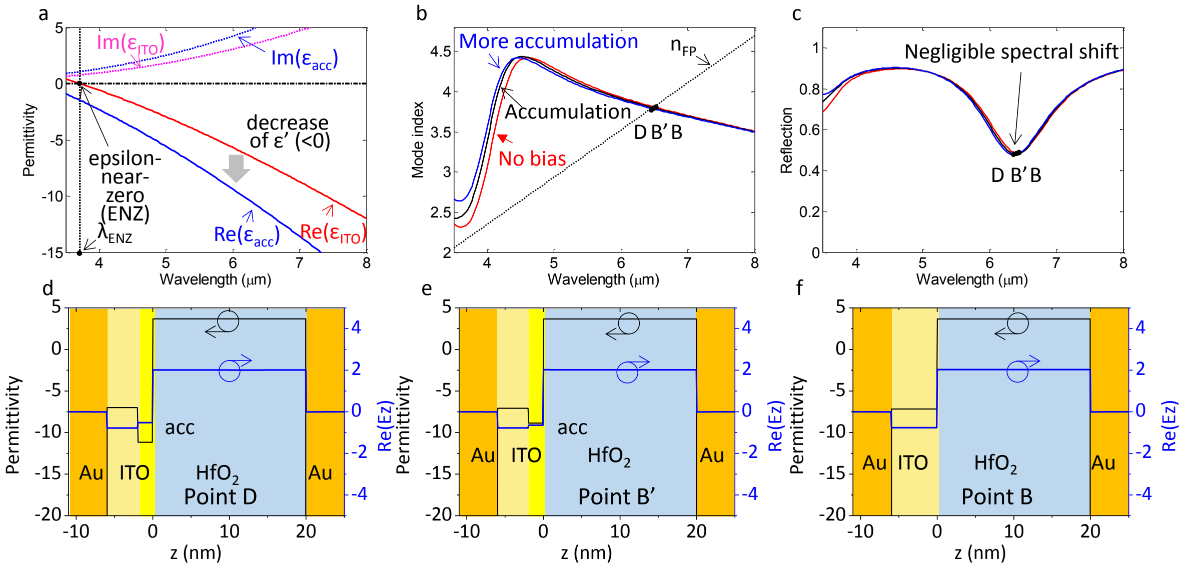

Figure S2.6. a, Permittivity of the ITO and the accumulation region for wide strips for which $\lambda_{\mathrm{res}} > \lambda_{\mathrm{ENZ}}$. **b,** Mode index of the gap plasmons for the cases of no bias, accumulation, and strong accumulation. The dashed black line denotes the Fabry-Pérot mode index n_{FP} for a strip width of 800 nm. **c,** Full-field simulation of the reflectance of a metafilm constructed from 800-nm-wide strips spaced at a period of 1,000 nm. **d,e,f,** Permittivity (black, the left y-axis) and the real part of E_z (blue, the right y-axis) distribution for Points D, B', and B in Figs. S2.6 **b** and **c**, respectively.

The aforementioned five cases, together with the depletion operation in the $\lambda_{\mathrm{res}} \approx \lambda_{\mathrm{ENZ}}$ regime in Fig. 2 in the main text, are summarised in Table S2.1. The key finding from this comprehensive study is that the depletion mode operation in the ENZ wavelength regime is most effective in achieving a high modulation of the absorption. In Supplementary

Information S6, we provide an experimental demonstration of this finding by showing the reflectance spectra for various strip widths that cover the case that $\lambda_{\mathrm{res}} < \lambda_{\mathrm{ENZ}}$, $\lambda_{\mathrm{res}} \approx \lambda_{\mathrm{ENZ}}$, and $\lambda_{\mathrm{res}} > \lambda_{\mathrm{ENZ}}$.

Table S2.1. Spectral shift of resonance

<table>
<tr><th></th><th>small width</th><th colspan="2">middle width</th><th>large width</th></tr>
<tr><td>reflectance dip</td><td>single dip</td><td colspan="2">double dips</td><td>single dip</td></tr>
<tr><td>resonance wavelength</td><td>$\lambda_{\mathrm{res}} < \lambda_{\mathrm{ENZ}}$</td><td colspan="2">$\lambda_{\mathrm{res}} \approx \lambda_{\mathrm{ENZ}}$</td><td>$\lambda_{\mathrm{res}} > \lambda_{\mathrm{ENZ}}$</td></tr>
<tr><td>depletion ($V_{\mathrm{ITO}} > V_{\mathrm{Au}}$)</td><td>redshift</td><td colspan="2">redshift   blueshift
(double dips-to-single dip)</td><td>blueshift</td></tr>
<tr><td>accumulation ($V_{\mathrm{ITO}} < V_{\mathrm{Au}}$)</td><td>blueshift</td><td>blueshift</td><td>(small) blueshift</td><td>(small) blueshift</td></tr>
</table>

S3. Depletion width

The extent to which the optical properties of ITO can be manipulated is limited by the achievable changes in the carrier density σ, and this quantity is directly related to the DC electric permittivity and the dielectric strength of the insulator as:

$$\sigma = \frac{\varepsilon_0 \varepsilon_{DC} E_0}{e}, \qquad \text{(Eq. S3.1)}$$

where σ, ε_0, ε_{DC}, E_0, and e denote the carrier density, electric permittivity in free space, the DC relative electric permittivity of the insulator, the applied electric field, and the electron charge, respectively. The dielectric breakdown in the HfO_2 we use is measured to be ~7.0 V across a 20-nm-thick insulator that we deposited (Supplementary Information S8). This corresponds to 3.5 MV/cm, which is in a conventional range of the HfO_2 layer, especially given that this layer is deposited on a metal rather than a semiconductor like Si. To avoid risk of dielectric breakdown, we used 5.0 V for the depletion operation. The DC electric permittivity ε_{DC} for HfO_2 is measured as 16. As a result σ is obtained as $2.21 \times 10^{13}/\mathrm{cm}^2$.

Meanwhile, the depletion width (t_{dep}) is theoretically predicted by:

$$t_{dep} = \frac{\sigma}{n}. \qquad \text{(Eq. S3.2)}$$

Here n is the electron density in the ITO layer, and is obtained from the Drude model and the plasma frequency (Supplementary Information S7) as $1.47 \times 10^{20}/\mathrm{cm}^3$. The estimated depletion width for 5 V across 20 nm of HfO_2 is thus given by 1.50 nm.

The maximum modulation ratio obtained from the experimental results above is 15% (Fig. 4b in the main text). Although this is a significant change, there still remains room for further improvements. One of the limitations is that we could not achieve a full depletion of the ITO layer; the estimated depletion width was 1.5 nm, whereas the total ITO thickness is 6 nm. One approach is thus to increase the depletion width. This can be achieved by using an insulating layer with a higher dielectric strength and a larger DC permittivity, or by using an ITO layer with the lower plasma frequency and a lower electron density *n*. HfO_2 exhibits a relatively high DC permittivity of 16, whereas SiO_2 and Al_2O_3 have 3.9 and 9.9, respectively. The dielectric strength of HfO_2, which was measured around 3.5 MV/cm in our configuration, would be further increased by using rapid thermal annealing for the reduction of defect states[S1,S2].

S4. Metafilm resonance and its link to the metal strip width

Figure S4.1 shows a map of the reflectance at normal incidence from an array of subwavelength Au strips implemented on top of the ITO/HfO_2/Au stack. Along the vertical axis is the strip spacing and along the horizontal axis is the strip width. The top Au strips have a thickness of 50 nm. The HfO_2 is a 20 nm-thick-layer and the bottom Au is semi-infinite. The illumination wavelength was fixed at 5 μm. To focus on the working principle of the optical absorber, we consider a case of in which full depletion is achieved in an ITO layer with the thickness of 6 nm. From Fig. S4.1, we note that the strip width determines the resonance and that the spacing only determines the depth of the reflectance feature. At strip widths of 760 nm, 2,212 nm, and 3,752 nm, the reflectance is strongly suppressed. This is because of the resonant absorption that occurs when the Fabry-Pérot resonance condition is satisfied. The absorption for the 760 nm width corresponds to the first-order resonance, whereas strip width of 2,210 nm and 3,750 nm support third and fifth order resonances.

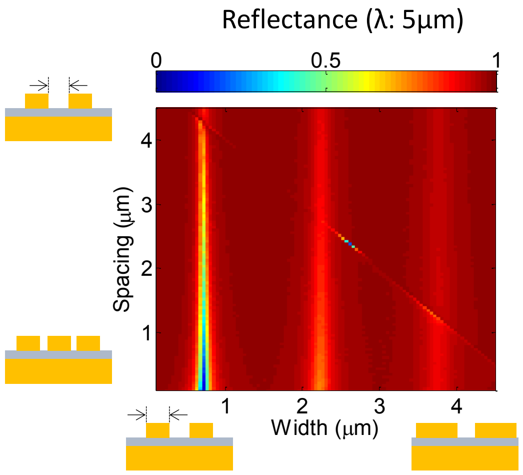

Figure S4.1. Reflectance for a metafilm consisting of subwavelength metallic strip cavities as functions of the strip spacing and width.

There is another kind feature on the reflectance map, which is a dip that occurs where the sum of the strip width and the spacing equals 5 μm. This is visible as a diagonal line running at a -45° angle through the reflectance map. This feature originates from a grating coupling effect by which the normally incident light is redirected into the horizontal plane with the plasmonic cavities. The structures dealt with in the main text and the following sections have strip widths and spacing values much smaller than those in the grating coupling regime; they are in the subwavelength metamaterials regime. Thus the devised configuration does not allow any higher order diffraction channels, and only the 0th order diffraction is allowed.

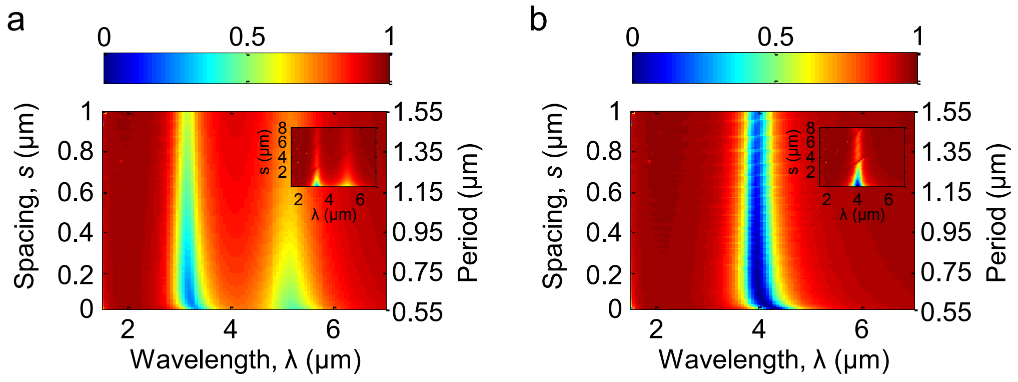

Figure S4.2. Reflectance as a function of wavelength and spacing for **a**, no-bias case and **b**, fully depleted ITO. The width is fixed as 550 nm.

To understand the dependence of the resonance wavelength on the strip width and its interaction with the materials resonance at λ_{ENZ}, we show in Fig. S4.2 the reflectance map as a function of wavelength and spacing for fixed width of 550 nm. The slight redshift around

the spacing below 100 nm can be ascribed to near-field interactions between neighbouring strip plasmonic cavities. At larger spacing than that, the spectral location of resonant wavelengths and the resultant reflectance minima are virtually independent of the spacing. Simulations for larger spacing (inset of Figs. S4.2a and S4.2b) show that this is valid until as long as no higher-order diffracted beams are created by the strip-array, i.e. in the subwavelength metamaterials regime.

S5. Equivalent model of the metamaterial

The proposed MIM antenna array has a deep-subwavelength period, and thus allows for only the 0th diffraction without higher order channel. Therefore, it is possible to apply metamaterial modelling to the geometry. Figure S5.1 a shows the schematic of the equivalent model of the metamaterial, where the top Au gratings/HfO_2/ITO layers are replaced with a homogeneous slab. The thickness of the slab is set to be equal to the sum of the thickness of top Au gratings, ITO layer, and HfO_2 layer.

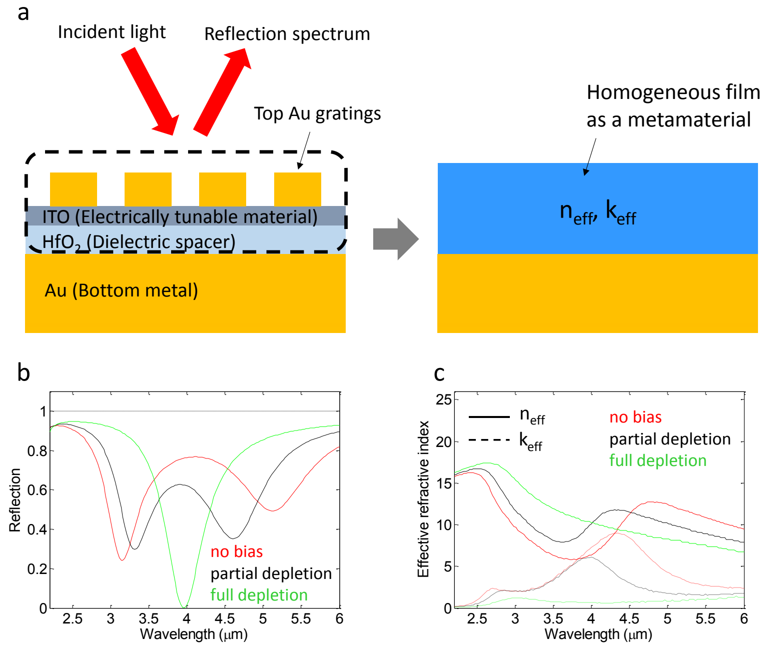

Figure S5.1. a, Metamaterial modelling of the deep-subwavelength antenna array as a homogeneous slab. **b,** Reflectance spectra calculated by RCWA. **c,** Extracted refractive index of the metamaterial n_{eff} and k_{eff}.

The reflectance spectrum calculated by using RCWA is shown again in Fig. S5.1 b for the sake of comparison. From the reflectance amplitude and phase we can extract the effective

refractive index of the metamaterial n_{eff} and k_{eff}. The results are shown in Fig. S5.1 c. We note that there can be a remarkable change in the optical properties of this equivalent metamaterial.

S6. Optical modulation for various grating widths

In addition to the strip widths of 490 nm, 600 nm, and 720 nm dealt with in the main text, we fabricated various structures with strip widths of 445 nm, 525 nm, 640 nm, 680 nm, 785 nm, and 865 nm. By examining how the reflectance spectra depend on strip widths, we can experimentally and numerically explore the dependence of the resonance wavelength on strip width. With that knowledge one can in turn optimise the absorption modulation efficiency at a target wavelength.

Figures S6.1 a to i show the reflectance spectra measured by the FT-IR microscope (top panel in each figure) and the full-field simulation results obtained by the RCWA (bottom panel in each figure). As in Fig. 4 in the main text, the red, green, and blue curves denote the no bias case, the positive electric gating (+5 V) for the depletion operation with the depletion width 1.5 nm, and the negative electric gating (-5 V) for the accumulation operation with the accumulation width 1.5 nm and the accumulation plasma frequency 1.15×10^{15} (rad/s). For insightful comparison, the *x*-axis is set the same in all figures. The ENZ wavelength is depicted as a vertical dashed black line in each figure. The thickness of the ITO and HfO_2 layer, the plasma frequency, the damping frequency, and the depletion width and the accumulation profile are the same as in those in Fig. 4 in the main text.

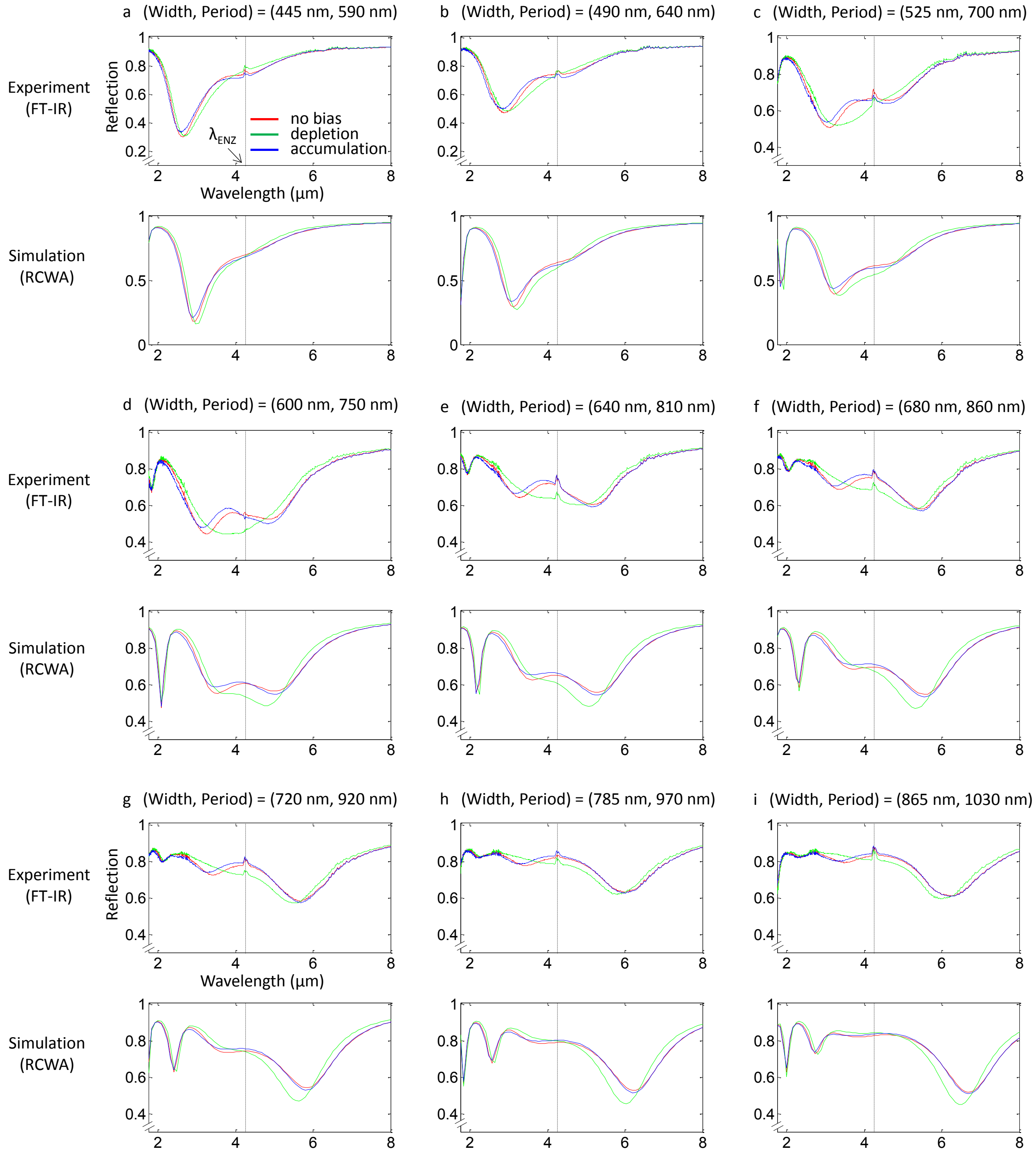

Figure S6.1. Reflectance spectra from various strip widths

We first note that, as the strip width is increased gradually, the resonance wavelength progressively redshifts. Single reflectance dips can be seen for metafilms with strips for which $\lambda_{res} < \lambda_{ENZ}$ or $\lambda_{res} > \lambda_{ENZ}$. The double reflectance dips occur when $\lambda_{res} \approx \lambda_{ENZ}$. The spectral shift of the reflectance spectra under the depletion and accumulation operations is in similar to the results shown in Fig. 4 in the main text and agrees well with Table S2.1. Finer increments of the strip widths display gradual changes, from which we can clearly see the difference of the

functional behavior of the reflectance spectra change as the strip width is increased. The simulation results show good agreement with the experiment.

S7. Extraction of the plasma frequency $\omega_{P,ITO}$

In order to extract the plasma frequency of the ITO $\omega_{P,ITO}$, reflectance spectrum spectra are taken from the ITO/HfO_2/Au stack (before strips are generated on top of this stack). The spectra were taken in a Fourier transform infrared (FTIR) microscope with a reflective lens having numerical aperture of 0.58 (the maximum incident angle around 35°). The thickness of the ITO and HfO_2 is 6 nm and 20 nm, respectively. The presence of the ITO layer perturbs the dispersion relation of the surface plasmon supported by the Au interface. At the plasma frequency the energy of the incident light is coupled to the surface plasmons supported by the air/ITO interface, which in turn leads to a decrease in the reflectance. This setup is similar to the Otto configuration[S3]. However, the incident angle is small, and thus the coupling from the incident light to the surface plasmons is not strong, resulting in very weak dip in the reflectance. To overcome this issue, we first measured the reflectance spectrum from the HfO_2/Au configuration as a reference signal, and then the signal from the ITO/HfO_2/Au was normalised by the reference signal.

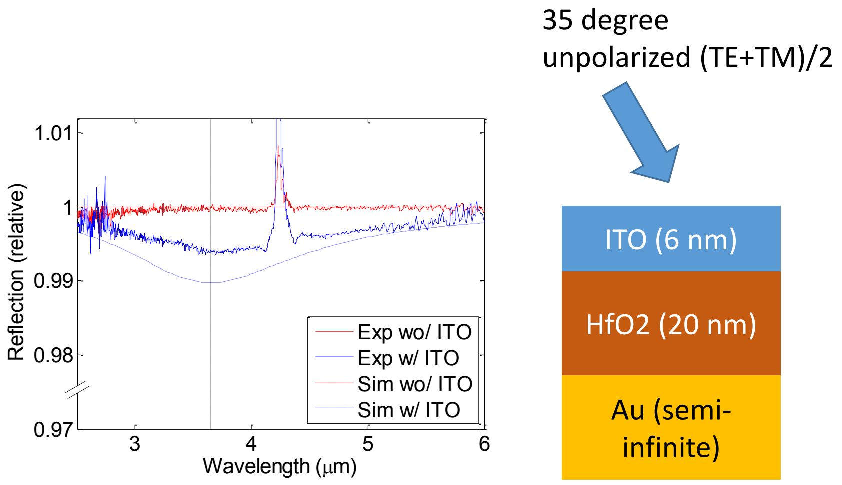

Figure S7.1. Relative reflectance spectrum of ITO/HfO_2/Au configuration for extraction of the ITO plasma frequency

Figure S7.1 shows the reference signal and the relative reflectance obtained by this method. Although the reflectance dip is shallow, it is still possible to detect a valley (solid blue curve). The transfer matrix method is used to calculate the relative reflectance spectra from the planar

HfO_2/Au and ITO/HfO_2/Au structures, which are denoted by the dashed red and dashed blue curves in Fig. S7.1. The simulation is performed for both polarisations of transverse electric (TE) and transverse magnetic (TM) light and the average of them is taken. Comparison of the experimental and simulation results show a slight discrepancy in the depth of the relative reflectance valley. This can be ascribed to potential scattering loss from surface roughness of the ITO film. We note that it still allows us to compare the spectral location of the center of the relative reflectance valley. By changing the plasma frequency $\omega_{P,ITO}$ in the simulation, we found that $\omega_{P,ITO}$ 1.02×10^{15} rad/s leads to the best fit. Under the damping frequency Γ of 2.6 $\times 10^{14}$ rad/s, the ENZ wavelength is obtained as 4.25 μm.

S8. Insulating properties of the HfO_2 on Au

The ability to electrically tune the optical absorption property of the presented device comes from controlling the carrier density in ITO. The electrical properties of the insulating layer is hence of critical importance. As the preparation of highly insulating oxides is more common on semiconductor materials, it is of value to provide a brief analysis of the insulating performance on Au and to this end we carried out current-voltage (IV) measurements where the voltage was ramped up and down in a single loop; Starting at 0 V, the voltage was first ramped up to 10 V and then decreased back to 0 V. The step size was 250 mV, and the time delay was chosen as 100 ms.

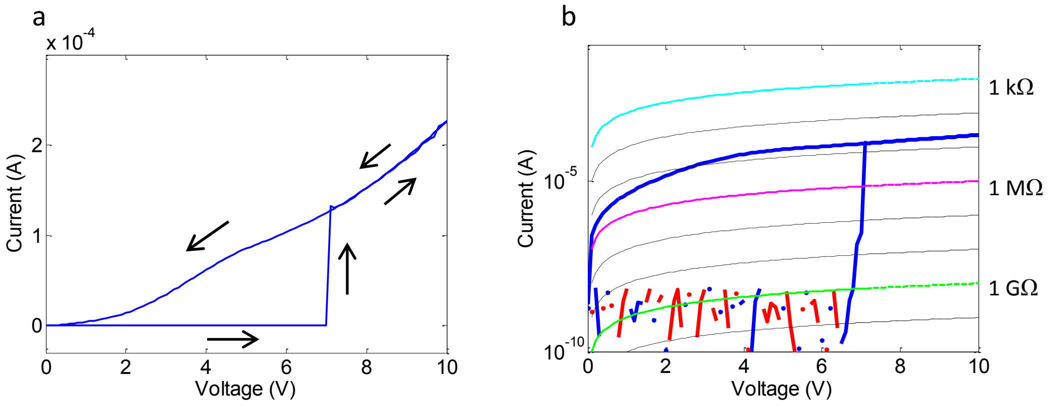

Figure S8.1. a, Current-voltage (IV) measurement on a 20 nm-thick-HfO_2 layer depicted on a linear voltage scale. **b,** The same data shown on a logarithmic scale to highlight the abrupt change in the current through the oxide layer (i.e. breakdown) The red curve corresponds to the current with a negative sign, which can be ascribed to the noise signal.

Figures S8.1 a and b show the experimental result. The insulating layer is 20 nm-thick-HfO_2, which is the same as that used in the paper. The arrows in Fig. S8.1 a are used to visualise the trajectory of voltage change. The current across the insulating layer is negligible when the voltage is less than around 7.0 V and it is not dependent on the voltage. It is noteworthy that at a voltage around 7.0 V, the current exhibits a significant and sudden jump up to ~100 μA. This comes from the dielectric breakdown of the insulating layer. As the voltage increases, the current increases and there is a linear dependence of the current on the applied voltage. This indicates that the HfO_2 layer became conductive, which is the result of the dielectric breakdown. The slope of the current upon the voltage directly tells us the resistance of the layer, which is around 56 kΩ. When the voltage reaches its maximum at 10 V, the current is 226 μA. As the voltage decreases back to zero, the current is decreases linearly.

To demonstrate the rapid rise in the current when breakdown occurs, the same data is also plotted on a logarithmic scale in Fig. S8.1 b. The cyan, magenta, and green curves denote the currents for constant resistance values of 1 kΩ, 1 MΩ, and 1 GΩ, and the dotted black curves between them correspond to an order of magnitude incremental step sizes. It is clearly shown that under the breakdown electric field of 7 V, the current is negligible and is independent of the applied voltage. The resistance is more than or in order of GΩ. As the applied voltage exceeds 7 V, the current steeps dramatically and the current depends on the voltage linearly with the ohmic resistance in tens of kΩ. It should be pointed out that we carried out this experiment for 20 other samples, and obtained similar breakdown electric field around 7.0 V. In order to prevent dielectric breakdown, we used maximum voltages of +5V in depletion mode. The current-voltage measurement is also performed for a negative electric bias to achieve accumulation, and the breakdown electric field was practically the same as for positive electric bias. Therefore, the voltage -5V was used for the accumulation.

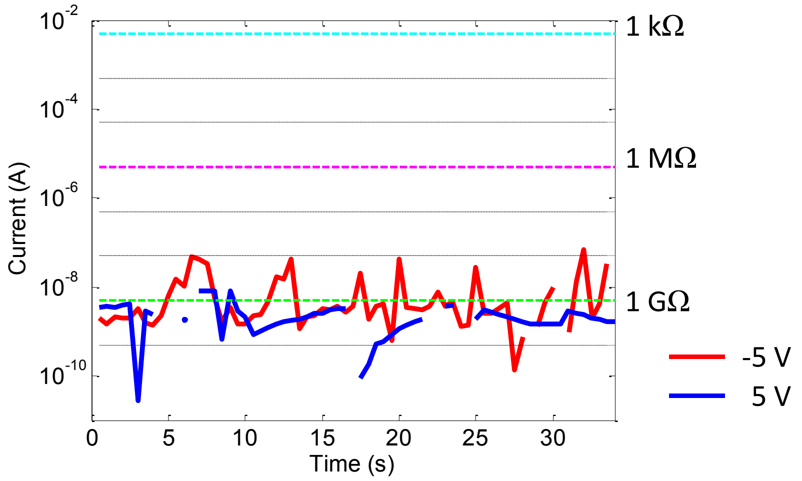

Figure S8.2. Current measurement during the electric gating

During the optical reflectance measurements in the FT-IR microscope, the current in the ITO/HfO_2/Au layer is recorded (Fig. S8.2). Both positive (5 V, blue curve) and negative (-5 V, red curve) electric bias for the depletion and accumulation are applied. The sign of the current in the negative bias is inverted. The discontinuity shown in Fig. S8.2 originates from the fact that the logarithm of current ($\log_{10}(I)$) does not display negative values. For comparison, current values I corresponding to resistances R of 1 kΩ, 1 MΩ, and 1 GΩ, are plotted for reference with cyan, magenta, and green horizontal lines, where ($I=V/R$).

S9. Polarisation dependence of the light absorption in the metafilms

In 1-D metallic gratings gap plasmon can only be excited when the electric field of an incident light wave is perpendicular to the direction of the strips in the metafilm (x-polarisation). The electric field in the parallel direction with the grating (y-polarisation) does not excite a gap plasmon for symmetry reasons as the electric field of the incident wave needs to match the longitudinal electric field of the gap plasmon. Since the absorption in our configuration comes from the Fabry-Pérot resonance of the gap plasmon, the reflectance dip can be found only in the x-polarisation. Figure S9.1 a shows the top view of the top Au gratings on the ITO layer together with the electric field direction depicted by the red (0°, x-polarisation), blue (30°), green (60°), and black (90°, y-polarisation) arrows.

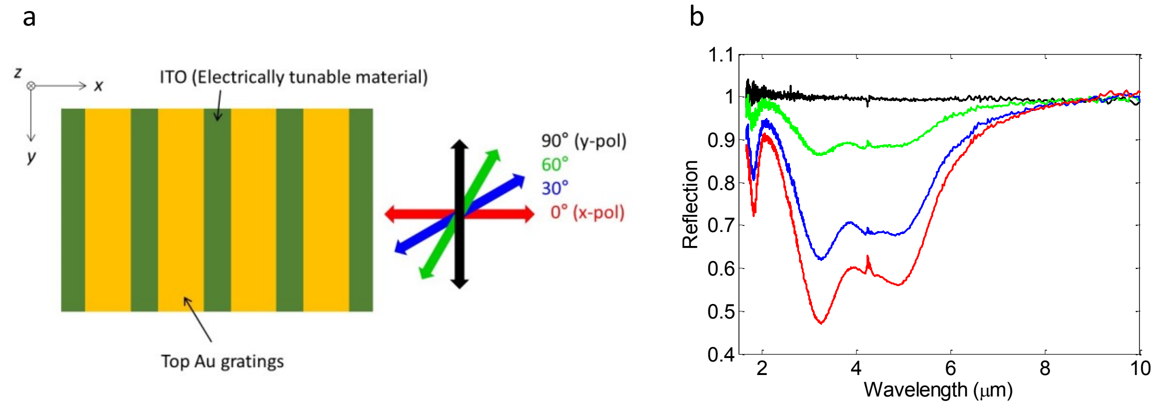

Figure S9.1. a, Top view of the 1-D top Au gratings on ITO/HfO_2/Au The red, blue, green, and black arrows denote the directions of the electric field of 0° (*x*-direction), 30°, 60°, and 90° (*y*-direction) polarisation states, respectively. **b,** Reflectance spectra taken for various polarisation directions as depicted in panel a.

The reflectance spectra of a metafilm with Au strips with a width 600 nm and a period of 750 nm are obtained with an FT-IR microscope and are shown in Fig. S9.1 b. Each curve corresponds to the polarisation state depicted in the same color in panel of this figure. The *x*-polarisation exhibits the strongest absorption as expected. In contrast, the *y*-polarisation does not display any change in the reflectance. This is because the *y*-polarisation does not excite the surface plasmon. The other polarisation states whose electric fields have the deviation angle 30° (blue) and 60° (green) with respect to the normal direction of the gratings show intermediate responses.

While the ITO layer afford effective optical modulation, it also perform a critical electrical role in affording effective charge inject and extracted. Because of the high conductivity of ITO, the top metal structures do not have to be continuous in order to electrically gate the devices. As such, one has great freedom in the choice of the size and shape of the metallic structures that can be put on top of the device. We have investigated patterns such as 2-D array of discs, squares, rectangles, and even further complicated geometries that may exhibit a desired optical absorption property. As an example, we show a 2-D arrays of disc with the diameter of 730 nm (inset of Fig. S9.2 a). The main advantage is that the resonance of the 2-D array of circular discs is insensitive to the polarisation angle of incident light. The localised surface plasmon itself is robust to the incident angle[S4,S5]. Due to the symmetry of the 2-D array it is not sensitive to changes in the azimuthal angle. Consequently we can achieve an omni-directional and polarisation-insensitive tunable optical absorber. Figure S9.2 a presents

the reflectance spectra from the 2-D array of Au structure on top of ITO/HfO_2/Au for various polarisation angles. The reflectance spectra remain almost the same under a change of the polarisation angle. This is different from the 1-D gratings, which exhibit a strong dependency on the polarisation angle. Figure S9.2 b shows the reflectance spectra under the electric gating without any polariser in the measurement setup. We can still obtain a significant change in the reflectance spectra by using depletion and accumulation.

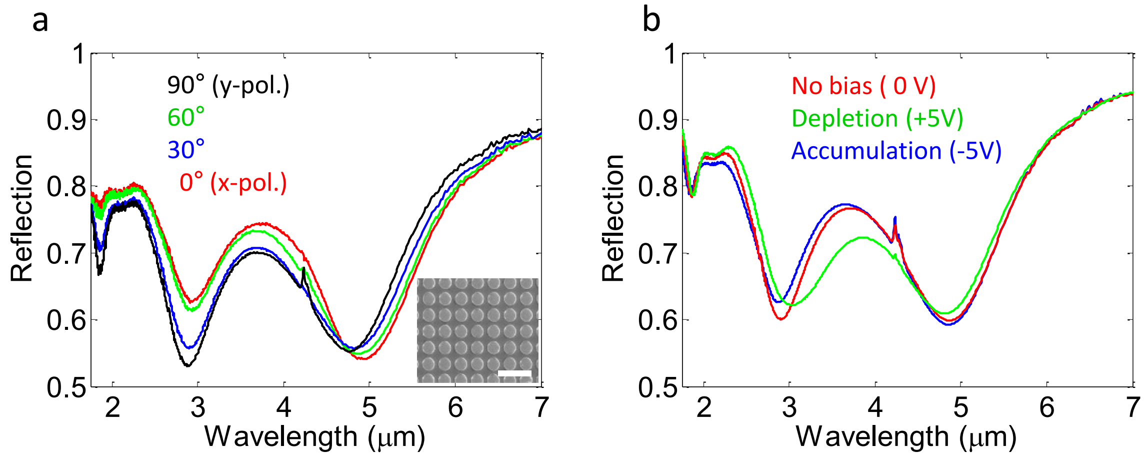

Figure. S9.2. Polarisation-independence of the tunable absorption in a metafilm consisting of a 2-D array of discs. a, Reflectance spectra of the 2-D array of Au discs on ITO/HfO_2/Au for various polarisation angles. The inset shows the scanning electron microscopy of the discs with the diameter 730 nm and a period 1,000 nm (scale bar: 2 μm). Because of the good conductivity of ITO, the top Au patterns do not need to be geometrically connected for electrical gating, affording a great flexibility for the design of the top metal patterns. **b,** Reflectance spectra under the electric bias: no bias (red, 0 V), the positive electric bias for depletion (green, +5 V), and the negative electric bias for accumulation (blue, -5 V).

In addition, these subwavelength configurations are expected to show a weak dependence of the light absorption to the incident angle as long as only one plasmon mode is excited and the excitation of grating orders beyond the 0th order are suppressed. To demonstrate this feature, we present in Fig. S9.3 the reflectance spectra for two different reflective objective lenses with numerical apertures (NAs) of 0.58 (red curve) and 0.30 (blue curve). The maximum incident angle for the lens with NA of 0.58 is 35°, whereas that of NA of 0.30 is 17°. The strip width and period is 600 nm and 750 nm, and the thickness of top Au, ITO, HfO_2, and the bottom Au layers are 50 nm, 6 nm, 20 nm, and 50 nm, respectively. There are two reflectance dips around 3.1 μm and 5.0 μm, originating from the Fabry-Pérot resonance. We note that there is a negligible change in the reflectance spectra. The two dips both correspond to the first-order resonance mode (but with a different gap mode index). The second-order Fabry-Pérot resonance mode can be seen around 1.9 μm. It does not appear for the normal

incidence since the normally incident light has only transverse electric component that only allows for the excitation of odd-ordered resonance modes (m = 1, 3, 5, ...) for symmetry reasons. As we use the objective lens with higher NA, which allows for larger incident angles, the even-ordered resonance modes (m = 2, 4, 6, ...) can be excited as well. Consequently, we observe that there is a negligible spectral shift at the resonance wavelength around 1.9 μm, and the increase in the incident angle results in the deeper reflectance dip.

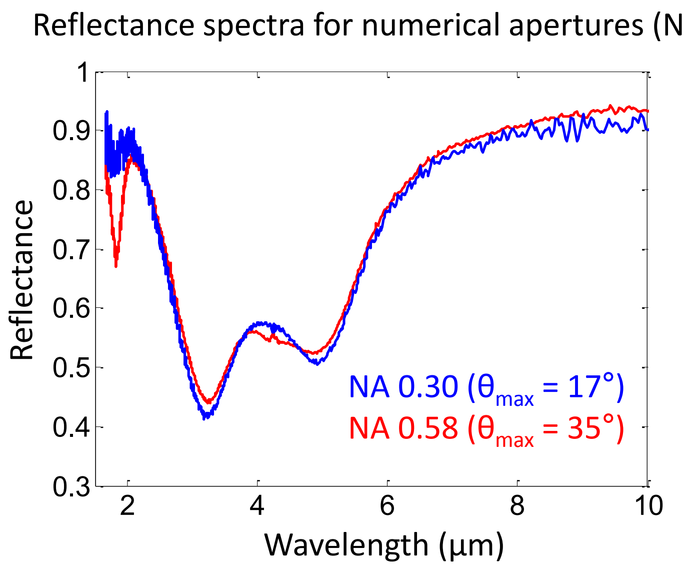

Figure S9.3. Angle independence of the light absorption in a metafilm consisting of metallic discs. Reflectance spectra from FT-IR measurement for numerical apertures (NAs) of 0.30 and 0.58.

S10. Refractive index of HfO_2 in infrared regime

The resonance condition of the electrically tunable optical absorber is strongly dependent on the mode index of the gap plasmon. It is therefore of critical importance to understand the optical materials properties of the dielectric spacer, the HfO_2 layer. Since we use a wide range of wavelengths from 1.7 μm to 10 μm, we need to take into account the dispersive nature of the optical materials properties of the HfO_2 into account for an accurate simulation. Figure S10.1 shows the measured real (n, the red curve) and imaginary (k, the blue curve) parts of the measured refractive index of the HfO_2 film deposited on a Si wafer by using atomic layer deposition. The real part of the refractive index decreases from 2.07 at 1.7 μm to 1.58 at 10 μm, which is quite significant. Beyond the wavelength of 10 μm, the loss in the HfO_2 layer

grows substantially.

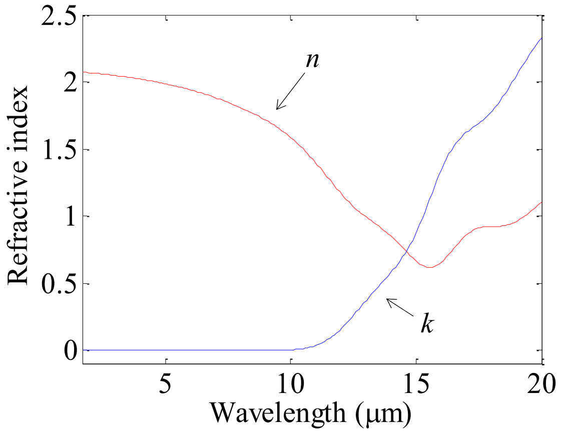

Figure S10.1. Optical constant of HfO_2 layer

References

S1. Lee, B. H. *et al.* Thermal stability and electrical characteristics of ultrathin hafnium oxide gate dielectric reoxidized with rapid thermal annealing. *Appl. Phys. Lett.* **76**, 1926 (2000).

S2. Zhan, N. *et al.* Effects of rapid thermal annealing on the interface and oxide trap distributions in hafnium oxide films. IEEE Conference on Electron Devices and Solid-State Circuits. 16–18 Dec. 2003.

S3. Otto, A. Excitation of nonradiative surface plasma waves in silver by the method of frustrated total reflection. *Zeitschrift für Physics* **216**, 398–410 (1968).

S4. Liu, N. *et al.* Infrared perfect absorber and its application as plasmonic sensor. *Nano Lett.* **10**, 2342–2348 (2010).

S5. Hao, J. *et al.* High performance optical absorber based on a plasmonic metamaterial. *Appl. Phys. Lett.* **96**, 251104 (2010).
